# A brain-enriched circRNA blood biomarker can predict response to SSRI antidepressants

**DOI:** 10.1038/s41380-026-03491-w

**Published:** 2026-02-17

**Authors:** Grigorios Papageorgiou, El Chérif Ibrahim, Victor Gorgievski, Gabriella Maxson, Evelyn Lozano, Eric Gordon, Antoine Lefrere, Marie-Julie Toupet, Philippe Courtet, Raoul Belzeaux, Jane Foster, Thomas Carmody, Roy H. Perlis, Madhukar H. Trivedi, Eleni T. Tzavara, Nikolaos Mellios

**Affiliations:** 1 Circular Genomics Inc., San Diego, CA USA; 2 University of New Mexico, Department of Neurosciences, Albuquerque, NM USA; 3 Aix-Marseille Univ, CNRS, INT, Inst Neurosci Timone, Marseille, France; 4 Fondation FondaMental, Créteil, France; 5 Université Paris Cité, Inserm, CNRS, HealthFex, F-75006 Paris, France; 6 Hôpital Sainte Marguerite AP-HM, Pôle de Psychiatrie, Marseille, France; 7 IGF, Université de Montpellier, CNRS, INSERM, Montpellier, France; 8 Department of Emergency Psychiatry and Acute Care, Lapeyronie Hospital, CHU Montpellier, Montpellier, France; 9 Department of Psychiatry, CHU de Montpellier, Montpellier, France; 10 University of Texas Southwestern Medical Center, Department of Psychiatry, Dallas, TX USA; 11 Center for Quantitative Health, Massachusetts General Hospital, Boston, MA USA; 12 Department of Psychiatry, Harvard Medical School, Boston, MA USA

**Keywords:** Neuroscience, Genetics, Predictive markers

## Abstract

Major Depressive Disorder (MDD) is a debilitating psychiatric disorder that is a leading cause of disability worldwide. Although treatment with antidepressants, such as Selective Serotonin Reuptake Inhibitors (SSRIs), has demonstrated clinical efficacy, the “trial and error” approach in choosing the most effective antidepressant treatment for each patient allows for only a subset of patients to achieve response to the first line of treatment. Circular RNAs (circRNAs), are highly stable and brain-enriched non-coding RNAs that are mainly derived from the backsplicing and covalent joining of exons and introns of protein-coding genes. They are known to be important for brain development and function, cross the blood-brain-barrier, and be highly sensitive to changes in both synaptic activity and neuronal receptor signaling. Here we present evidence that expression of the brain-enriched circRNA, CDR1as, is associated with symptomatic response to SSRI treatment, and regulated by serotonin and Brain-Derived Neurotrophic Factor (BDNF) receptor activity. We present data using circRNA-specific PCR in baseline whole blood samples from two independent cohorts, drawn from the Establishing moderators and biosignatures of antidepressant response in clinical care (EMBARC) and the Biomarkers of ANTidepressant RESponse (ANTARES) clinical studies, showing that before treatment CDR1as is differentially expressed between future symptomatic responders and non-responders to treatment with the SSRI sertraline. Additional data from naturalistic antidepressant response studies further highlight the association between CDR1as and antidepressant effects of SSRIs as a class. In addition, we show that CDR1as levels are altered following sertraline treatment in responders with the trajectory of change post-treatment associated with long-term remission. Furthermore, we report that levels of CDR1as in the blood can specifically predict remission with SSRI treatment, but not response/remission with Placebo or Bupropion treatments. Lastly, we provide evidence in animal mechanistic and neuronal culture studies, suggesting mouse Cdr1as is strongly regulated by 5-HT2A and BDNF receptor signaling. Taken together, our data identify a brain-enriched circRNA associated with known mechanisms of antidepressant response that can serve as a blood biomarker for predicting response and remission with SSRI treatment.

## Introduction

The prevalence of Major Depressive Disorder (MDD) has been steadily increasing over the last few decades [1] but has, since the COVID-19 pandemic, reached epidemic proportions within the US, with close to 30% of the adult US population now reporting to have been diagnosed with depression during their lifetime. Even though multiple classes of antidepressants, such as selective serotonin reuptake inhibitors (SSRIs), serotonin and norepinephrine reuptake inhibitors (SNRIs), and norepinephrine dopamine reuptake inhibitors (NDRIs), have demonstrated significant efficacy and safety, only a subset of patients significantly benefit from treatment [2, 3]. For example, fewer than 50% of patients with MDD experience significant symptomatic improvement, or response, with their first antidepressant treatment with remission rates being less than 30% [2, 3]. This trial-and-error approach leads to a large subset of patients undergoing multiple interventions, which results in significant delays in properly managing MDD, imposing a notable burden to individuals suffering with this devastating psychiatric disorder and resulting in significant costs to our healthcare system. Thus, there is a significant unmet need for developing reliable and robust biological biomarkers that could predict response to different antidepressants, thereby allowing for a precision medical approach to depression treatment.

Currently, the only widely available precision medicine tools to subjects with MDD are associated with DNA-based pharmacogenomics, which are designed to predict an individual’s genetic predisposition for metabolizing different psychiatric drugs, including antidepressants [4]. Although such approaches may identify individuals who differentially metabolize such medications, they are not able to achieve a direct prediction of response to antidepressant treatment. A known limitation of utilizing such genetic-based predictive biomarker approaches is the static nature of our genome versus the dynamic and adaptable nature of transcriptomic and proteomic biological signatures. However, the potential of any peripheral RNA and protein biomarker signatures to be capable of reliably predicting response to psychiatric drug treatments is significantly constrained by the fact that the vast majority of such molecules do not cross the blood-brain barrier (BBB) and are, thus, not directly associated with the molecular mechanisms that underlie psychiatric drug treatment response within the brain.

Circular RNAs (circRNAs) are a subtype of non-coding RNAs that are generated from non-canonical backsplicing and covalent joining of RNA, including exons and introns of protein-coding genes [5–9]. They have been found to be particularly enriched in the brain vs all other tissues and to be able to cross the BBB and to be readily-detectable in the blood [5–12]. Due to their unique structure, they are particularly resistant to exonuclease degradation, and are thus known to have robust inherent stability and much longer half-lives compared to linear mRNA molecules [11, 12]. CircRNAs are known to exert significant and diverse functional effects on gene expression by either sequestering microRNAs (miRNAs) and RNA-binding proteins (RBPs) or directly interacting with numerous RNA and protein partners to exert both transcriptional and post-transcriptional influence on a significant number of downstream gene targets [13]. Not surprisingly, given their abundance in neural tissue and significant upstream impact on gene expression, circRNAs, have been shown to be important for brain development, maturation, and function, and have been linked to a plethora of brain disorders [14–29]. Due to the above characteristics, circRNAs, thus, appear to hold significant molecular biomarker potential for better diagnosis and treatment of psychiatric and neurological disorders [27, 28].

Multiple studies [8, 10, 22, 30–33] have determined that CDR1as is a conserved circRNA that is particularly abundant in human and other mammalian brain tissues and is significantly enriched within cortical neurons. Previous mechanistic studies have also shown that CDR1as is a significant regulator of activity-dependent neuronal gene expression, excitatory neurotransmission, synaptic plasticity, and cognitive function [8, 10, 22, 30–33]. Here we provide evidence that CDR1as expression in whole blood is significantly associated with response to sertraline treatment in two independent clinical biomarker studies of antidepressant response and provide additional data from naturalistic studies linking CDR1as expression to antidepressant response of SSRIs as a class. We highlight the specific nature of CDR1as at predicting response to SSRI treatment in baseline whole blood samples of patients with MDD and uncover a dynamic expression profile post treatment that is associated with possibility of remission. Furthermore, we show that the expression of this circRNA in mouse brain is regulated by serotonin and Brain-derived neurotrophic factor (BDNF) receptor activity. Taken together, our data provide evidence of a biomarker that is predictive of response to SSRI antidepressant treatment and is significantly associated with known molecular mechanisms of antidepressant response.

## Materials and methods

### Clinical study cohorts

**Fig. 1:**
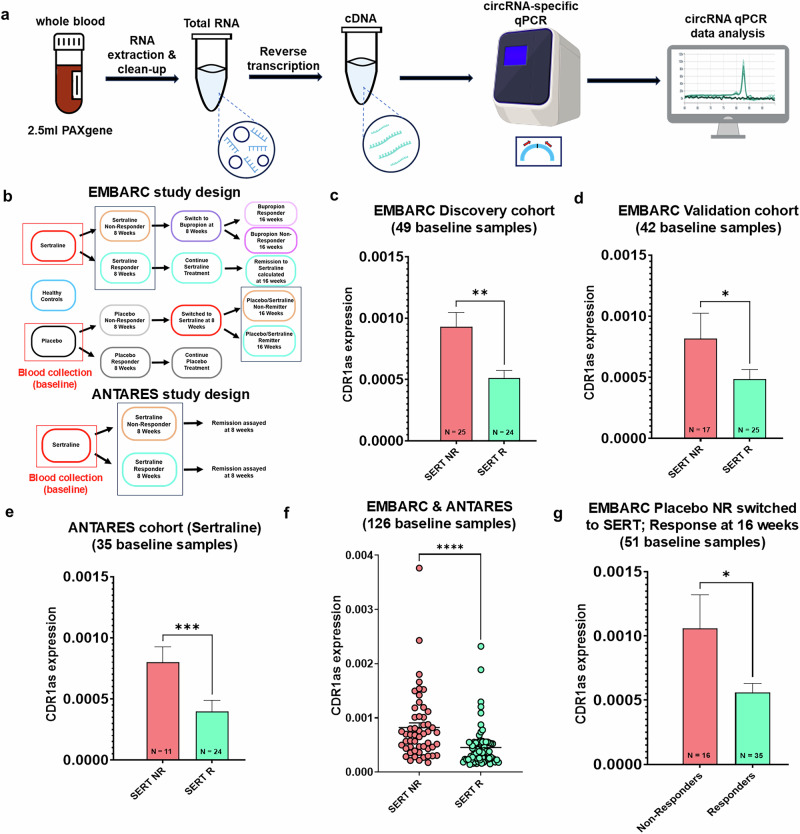
Baseline blood CDR1as circRNA levels predict response and remission following sertraline treatment. Schematic of the experimental design (**a**). Human whole blood samples (PAXgene RNA IVD tubes) were obtained, subsequent RNA extraction and RNA-cleanup was performed followed by reverse transcription and circRNA-specific qPCR and quantification (see also Materials and Methods). Schematic of the EMBARC and ANTARES antidepressant response studies (**b**). Rectangular shape indicates the study arms relative to the graphs included in this figure. Baseline whole blood CDR1as expression levels in SERT-R and SERT-NR in discovery (**c**) and replication (**d**) experiments with the EMBARC study and withing the ANTARES clinical study (**e**). Baseline whole blood CDR1as levels in the totality of the sertraline baseline samples from both clinical cohorts (**f**). Baseline whole blood CDR1as levels in Placebo non-responder patients who were then switched to sertraline at 8 weeks and were examined for response to sertraline treatment at 16 weeks (patients separated to Remitters and Non-Remitters to treatment) (**g**). For c-e, *p < 0.05, **p < 0.01, ***p < 0.001, ****p < 0.0001, based on two-tailed Mann-Whitney test. For g, *p < 0.05, based on one-tailed Mann-Whitney test. Each graph is shown as Mean + SEM with the number of individual biological samples included within each graph.

We utilized baseline and post-treatment whole blood PAXgene samples and clinical information drawn from the multi-site NIMH-funded Establishing Moderators and Biosignatures of Antidepressant Response in Clinical Care (EMBARC) completed study (https://clinicaltrials.gov/study/NCT01407094) [34] and the ongoing Biomarkers of ANTidepressant RESponse (ANTARES) study supported by ERA-NETs European Funding for Neuroscience Research (NEURON) fund (https://www.neuron-eranet.eu/projects/ANTaRES/; clinical trials number NCT05568823; ethical approval: Comite De Protection Des Personnes Ile De France III; Dossier n°: 2022-A00352-41; Réf. CPP: 4091; Réf. RIPH2G: 22.01705.000148). Both clinical studies were specifically designed to uncover biomarkers for prediction of response to antidepressant treatment and included whole blood samples and detailed clinical information before (baseline) and after treatment with the SSRI sertraline (see Fig. 1a, b). Response to treatment was defined as improvement of 50% or more in depression severity scores derived from clinician-rated scales, namely the Hamilton Depression Rating Scale (HAMD-17; for EMBARC) and the Montgomery–Åsberg Depression Rating Scale (MADRS; for ANTARES). Remission in EMBARC was assessed at 8 and 16 weeks after treatment and defined as a HAMD-17 score ≤ 7. Remission in ANTARES was assessed at 8 weeks after treatment and was defined as a MADRS score ≤ 9. In the context of the ongoing recruitment of the ANTARES study, all the PAXgene samples (originating from patients treated with sertraline and followed for 8 weeks) that were available as of March 2025 have been included in the analysis and presented in this study. For calculation of effects of life stress and anxiety the Holmes-Rahe Life Stress [35] questionnaire score, as well as the prevalence of Social Anxiety and of Generalized Anxiety Disorder according to the MINI diagnostic interview [36] were used. In addition to the sertraline arm of the study, EMBARC included a placebo-treated group (see Fig. 1a, b). Patients who did not respond to Placebo treatment at 8 weeks, were switched to sertraline and remission was assayed at 16 weeks as described above (Fig. 1a, b). Patients who did not respond to sertraline at 8 weeks, were switched to treatment with Bupropion and remission was assayed again at 16 weeks (see Fig. 1a, b). For EMBARC, one PAXgene whole blood sample per patient was available at baseline (before-treatment) and at 8 weeks after treatment (for a subset of the patients). For ANTARES PAXgene whole blood samples were obtained from patients before initiation of antidepressant treatment with sertraline as well as 3 days and 2 weeks after initiation of the treatment. Additional whole blood PAXgene samples were derived at random post-treatment intervals from University of Texas Southwestern (UTSW) as part of the Resilience in Adolescent Development (RAD; https://clinicaltrials.gov/study/NCT03458936) and Dallas 2 K: A Natural History Study of Depression (D2K; https://clinicaltrials.gov/study/NCT02919280 ?term=Dallas%202 K&rank=1) naturalistic depression and bipolar disorder studies. For the UTSW Naturalistic studies, there were no available baseline and post-treatment MADRS or HAMD-17 questionnaire data to determine response and remission, but data from the Patient Health Questionnaire-9 (PHQ-9) were available for the majority of the patients at random post-treatment intervals and were used to determine patients that are faring well under current treatment with the SSRIs fluoxetine, sertraline, citalopram, and escitalopram, which were defined as having PHQ-9 score of 14 or lower corresponding to: 6 patients with Minimal Depression (PHQ-9: 0–4), 8 patients with Mild Depression (PHQ-9: 5–9), and 3 patients with moderate Depression (PHQ-9: 10–14). The average PHQ-9 score for these 17 adult patients characterized as faring well under SSRI treatment for which whole blood CDR1as expression data were available was 6.1. Data on CDR1as whole blood levels were also obtained from a total of 10 adult patients who were treated with SNRIs (duloxetine, venlafaxine, desvenlafaxine), but no stratification was available based on PHQ-9 scores. In addition, whole blood CDR1as expression was collected from a subset of 18 adult patients with diagnosis of bipolar disorder, as well as 8 adolescent patients (ages 13–17), out of which 4 were characterized as faring well under treatment using the same criteria mentioned above for adult patients. In all clinical studies, blood was collected in the morning after overnight fasting. For the sample stability study, 1-2 PAXgene whole blood samples were obtained from 40 healthy Controls (Discovery Life Sciences) either earlier in the morning after overnight fasting (fasting group), later in the morning after having breakfast (breakfast group), or in the afternoon after having lunch (lunch group) to test for effects of fasting/eating and time of the day.

### RNA extraction from human blood samples and RNA cleanup

RNA isolation from PAXgene whole blood samples was done using the PAXgene Blood RNA kit 50, v2 IVD, PreAnalytiX (Qiagen, Hilden, Germany) following the manufacturer’s supplied protocol with few modifications. RNA quality (A260/280 and A260/230) as well as concentration of isolated total RNA was assayed through NanoDrop One Spectrophotometer (Thermo Fisher Scientific, Waltham, MA). Following RNA extraction, RNA was further processed using the Monarch RNA cleanup Kit (New England Biolabs, Ipswich, MA). Protocol steps were executed according to manufacturer’s instructions with slight modifications, and the RNA was finally eluted in a total volume of 40ul of RNase/DNase free water. RNA quality and concentration were then again assayed using NanoDrop One Spectrophotometer (Thermo Fisher Scientific). Samples with a concentration higher than 50 ng/ul and 260/280 ratio higher than 1.8 were used for any downstream RNA applications.

### Reverse transcription and circRNA-specific PCR

Reverse transcription of 500 ng of total RNA was performed using the SuperScript IV First-Strand Synthesis System (Thermo Fisher Scientific) per the manufacturer’s instruction and via use of random hexamers. Reverse transcription was performed using the Veriti Dx 384 well Thermal Cycler, designed for clinical use (Applied Biosystems, by Thermo Fischer Scientific). cDNA was then diluted 1:8 with RNase-free H2O. Quantitative real time PCR (qRT-PCR) was done using PowerUp SYBR Green Master Mix (Thermo Fisher Scientific) along with custom designed, validated, and sequence-verified circRNA primers (see Supplementary Table 1). Primer slopes were run for each new circRNA primer and circRNA qRT-PCR products were run on an agarose gel and sequences of each PCR product were further validated by sequencing. At the end of each qPCR amplifications plots and melt curves (ΔRn vs cycle per well) were automatically calculated by the Quant Studio 7 Pro qPCR instrument (Thermo Fisher Scientific). Relative circRNA levels were quantified directly from the Ct average’s (average of triplicate Ct values) as shown before [16, 19]. A reportable Ct average range was set up for human CDR1as a priori (27.5 ≤ Ct average < 33) and samples with average Ct of less than 27.5 or more or equal to 33 did not pass Quality Control (QC) and were considered inconclusive. Although housekeeping gene expression in the blood could be useful as a potential normalizer, we were not able to identify housekeeping genes that could match the stability and reproducibility of our circRNA blood biomarker. Levels of lncRNA CYRANO, also known as OIP5 antisense RNA 1 (OIP5-AS1) and LINC000632 were determined in the same cDNA used for circRNA detection using specific Taqman assays (see also Supplementary Table 1). No data exclusion was allowed for any clinical samples passing the pre-determined QC and reportable range parameters, even if they could be statistically identified as outliers. All PCR experiments for clinical samples were done with the experimenter being blind to the identify of each sample.

### Venous blood leucocyte extraction and RNA isolation

Eligible study participants were part of the replication cohort of a larger multi-site naturalistic study of adults diagnosed with MDD (ClinicalTrials.gov with ID: NCT02209142; Comite De Protection Des Personnes Sud Mediterranee V) [37]. 8-9 ml of whole blood was collected in EDTA tubes and processed as described before [37]. Leukocytes were captured using the LeukoLOCKTM filter (Thermo Fisher Scientific). Briefly, the filter with the captured leukocytes was washed with RNAlater® and then moved to a –80 °C freezer. TRI reagent (Thermo Fisher Scientific) was used to lyse the cells, which were then mixed with Bromo-3-chloro-propane (Sigma Aldrich, St. Louis, MO). Following centrifugation, total RNA was precipitated with ethanol and further purified and washed 0.1 mM EDTA. Total RNA was then treated with DNase using the DNA-freeTM kit (Thermo Fisher Scientific). Samples from patients with MDD, Bipolar Disorder, and unaffected controls were included in this study.

### RNA extraction and miRNA quantification in whole blood samples

For detection of miR-7a-5p in whole blood of patients from the ANTARES cohort, 20 ng of total RNA extracted as shown above was used and converted to cDNA using the TaqMan® Advanced miRNA cDNA Synthesis Kit (Applied Biosystems, ThermoFisher Scientific), following the manufacturer’s instructions. Quantifications by real-time qPCR were performed with the Advanced miRNA assays, (Supplementary Table 1) selected from the web portal of ThermoFisher, and the TaqMan® Fast Advanced Master Mix (Applied Biosystems, ThermoFisher Scientific) on a QuantStudio 7 Pro thermocycler (ThermoFisher Scientific). The level of expression of miR-7a-5p was normalized by a reference miRNA, miR-30d-5p (previously shown to be stable in white blood cells of MDE patients and controls [38]). Each reaction was run in triplicate. The raw qPCR results were mined using ThermoFisher’s cloud-based RQ software with baseline and threshold set manually.

### RNA extraction and circRNA quantification in cell cultures and mouse brain tissue

For RNA isolation of cultured cells or brain tissues extracted from mouse brains, RNA was isolated using the miRNeasy RNA isolation kit (Qiagen) following the manufacturer’s supplied protocol. RNA quality as well as concentration of isolated total RNA was assayed through Nanodrop 2000 spectrophotometer (Thermo Fisher Scientific), with all samples passing the quality control measurements (A260/230 and A260/280). Reverse transcription of total RNA (100 ng or 500 ng; depending on initial RNA concentration) and circRNA-specific PCR was done as described above [16, 19].

### SH-SY5Y Human neuroblastoma cell line treatments

The SH-SY5Y epithelial human neuroblastoma cell line was purchased from ATCC (Manassas, VA; product code CRL-2266™). Their morphology and viability were observed daily under the microscope. After reaching passage #7, SH-SY5Y cells were plated in a 24-well plate at a concentration of 100,000 cells per well. 48 h after been plated, SH-SY5Y cells were treated with ANA-12, an industrial TrkB receptor antagonist, purchased from Tocris Bioscience (Bristol, United Kingdom; catalog no 4781). ANA-12 was dissolved in DMSO and to compensate for the effects of DMSO in cells viability, a negative control / vehicle group was included with the same concentration of DMSO. 24 h after treatment, cells were harvested and processed as described above using the miRNeasy RNA isolation kit (Qiagen).

### Primary mouse cortical neuron pharmacological treatments

Mouse cortical neuronal cultures were purchased from Thermo Fisher Scientific – Catalog number: A15585. Neurons were isolated and cryopreserved from C57BL/6 mice at embryonic day-17. Neurons were plated at a density of 4 × 10^^4^ cells/12-mm at a coverslip coated with poly-Ornithine on a 24-well plate. Neurons were allowed to adhere for about 30 min before adding 500uL of their respective media. Neurons were fed by replacing half the plating media volume with fresh media every third day. A media of a combination of Neurobasal Plus, 1XB27 Plus, 2 mM Glutamax and 5% Pen/Strep was used (all items purchased from Thermo Fisher Scientific). At DIV13, when neurons reach their maturation state (as observed with microscopy), pharmacological treatments were conducted as mentioned below. All the small molecule inhibitors were dissolved in DMSO. A negative control / vehicle group was included with the same concentration of DMSO. 24 h after treatment, neurons were subjected to RNA extraction as shown above.

CREB and CREB-CBP pharmacological inhibition: For restricting the interaction of CREB with its co-transcriptional activator CBP, a specific KIX-KID interaction inhibitor was used (CAS 92-78-4; Millipore/Sigma Aldrich) and was added at a concertation of 2.5 µM.

Small molecule kinase inhibitors: To examine which intracellular neuronal induced kinase can alter the levels of our circRNA of interest, various candidates were tested in primary cortical neurons. All pharmacological agents were added at DIV13 (dissolved in DMSO) and were purchased from Tocris Bioscience corporation. H 89 dihydrochloride (Cat. No. 2910), a selective PKA inhibitor was added at a dose equal to 120 nM. GF 109203X (Cat. No. 0741) was used to inhibit PKC activity at a dose equal to 0.2 µM. FR 180204 (Cat. No. 3706) was used for ERK1,2 inhibition at a dose of 0.3 µM.

### In vivo Pharmacological treatments in animals

All animal protocols complied with French and European Ethical regulations. The experimental protocols were approved by the local Ethical Committee (Comité d’ éthique en experimentation animale Charles Darwin N^o^5; protocol number #23824). C57BL/6 J 5-week-old male mice were purchased from Charles River (France). Mice were housed in cages of 5, at 22 ± 1 °C, and a 12-h light-dark cycle. Humidity levels were between 45 and 55%. Food and water were made available *ad libitum*. Pharmacological treatment was conducted during the second half of the light phase. At 6 weeks, mice were assigned pseudo-randomly (no randomization) to one of the treatment groups detailed below. For sub-chronic inhibition of the serotoninergic and dopaminergic system, mice were treated for 14 days with either a vehicle solution (control group), a selective 5HT-2AR antagonist (MDL100907, 2 mg/kg), and a selective D2-R antagonist (sulpiride, 25 mg/kg). For sub-chronic inhibition/activation of the glutamatergic system, mice were treated for 7 days with either a vehicle solution, a NMDAR antagonist (MK801, 0.3 mg/kg), a mGluR5 positive allosteric modulator (CDPPB, 10 mg/kg). Drugs were purchased from Sigma-Aldrich: MDL100907 (M3324), MK-801 (M107) and Tocris: CDPPB (#3235), Sulpiride (#0894). Agents were dissolved in a vehicle solution that was composed of 80% saline, 10%DMSO, 10% Cremophor and then were administered via intraperitoneal injection in mice daily at a volume equal to 10 mL/kg. No statistical methods were used to pre-determine sample sizes, but our sample sizes (n = 10-12 per group) were similar to those generally employed in the literature for the same paradigms and experimental design; similarly the selection of the vehicle, administration route, and the doses administered were based on the literature and our experimental experience [39]. Prior to sacrificing the animals, an intraperitoneal (i.p.) injection of pentobarbital (Euthasol; 400 mg/kg) was used to fully anesthetize them. Brain was collected and was immediately frozen in dry ice. For RNA extraction, reverse transcription, and qPCR procedures, the protocols used were described in their respective paragraphs earlier in the beginning of this section. qPCR experiments were run by an experimenter blinded to the pharmacological treatment of the animals.

### Cohort information and study design for the sertraline arm of the clinical studies

All clinical groups were matched or balanced for numerous clinical and demographic components, including age, sex, depression severity. For the sertraline arm of the EMBARC study, one PAXgene tube from each of the 103 patients was obtained in total. These were randomly divided into two cohorts (EMBARC discovery and validation cohorts) based on the de-identified sample ID and timing of RNA extraction (RNA extraction was performed in two different time points). For the discovery cohort 57 samples were processed with 49 samples passing QC (8 samples failed QC due to average Ct values being outside the predetermined reportable range of 27.5–33.0). For the validation cohort 46 samples were processed with 42 samples passing QC (4 samples failed QC due to Ct values being outside the predetermined reportable range). For the ANTARES clinical study, two PAXgene samples from 38 patients with MDD were provided before treatment with sertraline (baseline). Originally 33 out of the 38 ANTARES baseline samples passed QC (4 inconclusive samples outside the reportable range). Data from an additional two samples that originally failed QC in the first PAXgene tube experiments were derived by utilizing the second PAXgene tube, thus increasing the percentage of samples passing QC to 92.1% (35/38) for the ANTARES cohort compared to 88.3% observed in EMBARC, where one PAXgene tube was available per sample. For analysis of accuracy, positive predictive value (PPV), negative predictive value (NPV), sensitivity, and specificity (see also below in statistical analysis), a single Ct average cutoff of 30.7134 was selected for CDR1as based on the EMBARC discovery cohort (ideal Ct cutoff for highest sensitivity and specificity in the discovery cohort via use of the Graphpad Prism’s ROC curve calculator), and kept locked for the EMBARC validation and ANTARES cohorts and any additional clinical cohorts and analyses (Ct > 30.7134 = Responder to sertraline or SSRIs, Ct ≤ 30.7134 = Non-Responder to sertraline or SSRIs).

### Statistical analysis

The Prism statistical analysis software (Graphpad Software Inc) was used for all statistical analysis. Examination of normality of data distribution was carried out using the Shapiro-Wilk normality test. G*Power 3.1.9.7 was used to determine the required sample size for our clinical studies. A total sample group size of 52 was calculated based on effect size of 0.80 and power of 0.80 (a = 0.05). For comparisons between two groups where the direction of change was unknown a two-tailed *t*-test (normal distribution of data) or two-tailed Mann-Whitney test (non-normal distribution of data) was used. For comparison between two groups where the direction of change was already hypothesized, one-tailed *t*-test or Mann-Whitney tests were used. For comparison of more than two groups a one-way ANOVA with post-hoc Dunnett’s multiple comparisons test (comparing all to a control group) was used for data with normal distribution, while a Kruskal Wallis ANOVA with Dunn’s multiple comparisons test (comparing all to a control group) was used. For comparisons between all three groups, a one-way ANOVA with Tukey’s multiple comparisons test was used. For comparison between 8 week and baseline expression data within the same patients a two-tailed paired *t*-test was used. For comparisons between percentages of patients that were characterized as high or low likelihood to respond to SSRI or SNRI treatment in the naturalistic UTSW cohort, two-tailed Chi-square with Yates’ correction was used. Prism’s ROC curve calculator was used for calculating AUC and related p-values. The MedCalc statistical software was used for calculating PPV, NPV, Sensitivity, Specificity, and accuracy values.

## Results

### Blood expression levels of CDR1as circRNA are reliable and specific predictors of response to sertraline treatment at baseline

To determine whether brain-enriched circRNAs could serve as blood biomarkers for predicting response to antidepressants, we extracted RNA from whole blood PAXgene samples from two independent antidepressant response biomarker studies: the EMBARC [34] and ANTARES studies. We then performed RNA clean-up and reverse transcription, followed by qPCR (Fig. 1a) with validated circRNA-specific primers aimed at the unique circRNA backspliced junction. We chose to measure the expression of CDR1as, a circRNA that is particularly enriched in human brain vs multiple other tissues (Supplemental Fig. 1a–c), which has been shown by numerous studies to be important for neuronal gene expression, brain, function, and cognition [8, 10, 22, 30–33]. We hypothesized, that given the well-established links between neuroplasticity and both the pathogenesis of depression and the potential mechanisms underlying its treatment [40–42], that a brain-derived circRNA strongly linked to synaptic function and cognition and readily detectable in the blood would be a great candidate as a biomarker of antidepressant response. We initially focused on the sertraline arm of the EMBARC study (Fig. 1b) and measured the expression of CDR1as at baseline whole blood samples (before treatment). Clinical response to sertraline was then determined as 50% or greater improvement in the HAMD-17 depression severity score at 8 weeks after treatment (Fig. 1b). In the EMBARC cohort, we found that baseline CDR1as whole blood levels exhibited a significant difference in patients who responded to sertraline treatment (SERT-R) compared to patients who did not respond (SERT-NR) (Fig. 1c–e). Specifically, in our discovery cohort of 49 baseline blood samples from patients treated with sertraline we found a significantly higher expression (close to 1.80-fold) in CDR1as in SERT-NR vs SERT-R (Fig. 1c). We then quantified CDR1as in another set of 42 baseline samples from the EMBARC cohort (replication cohort) and uncovered a similar upregulation (close to 1.70-fold) in SERT-NR vs SERT-R samples (Fig. 1d). Looking at the totality of the sertraline baseline samples (N = 91), we observed a clear distinction between SERT-R and SERT-NR based on baseline CDR1as blood expression (p = 0.0003 based on two-tailed Mann Whitney test). Such results were specific to this circRNA, since expression levels of circTULP4 and circCRY2, two other brain-enriched circRNAs investigated in our study in the totality of the EMBARC cohort (Supplemental Fig. 2a, b), did not exhibit any difference between SERT-R and SERT-NR at baseline (Supplemental Fig. 2c, d).

To validate these findings in an independent clinical cohort, we repeated these experiments in 35 whole blood PAXgene samples from the ongoing European ANTARES antidepressant response biomarker study. Our results showed a significantly higher expression of CDR1as (2-fold) in baseline whole blood samples of patients who did not respond to sertraline treatment vs SERT-R (Fig. 1e), just as it was observed in the EMBARC cohort. Looking at the collective data from both clinical cohorts (N = 126 patients in total), we observed a robust significant difference in baseline CDR1as levels between SERT-R and SERT-NR (Fig. 1f; p < 0.0001 with on average 1.82-fold higher expression in SERT-NR vs SERT-R). Of note, looking at the raw average Ct data for CDR1as, similar significant differences were observed between SERT-R and SERT-NR in both the EMBARC and ANTARES clinical cohorts (Supplementary Fig. 3a–d; p = 0.014 for EMBARC discovery cohort, p = 0.0410 for EMBARC validation cohort, p = 0.0007 for ANTARES validation cohort, and p < 0.0001 for all samples together based on two-tailed Student’s *t*-test).

**Fig. 2:**
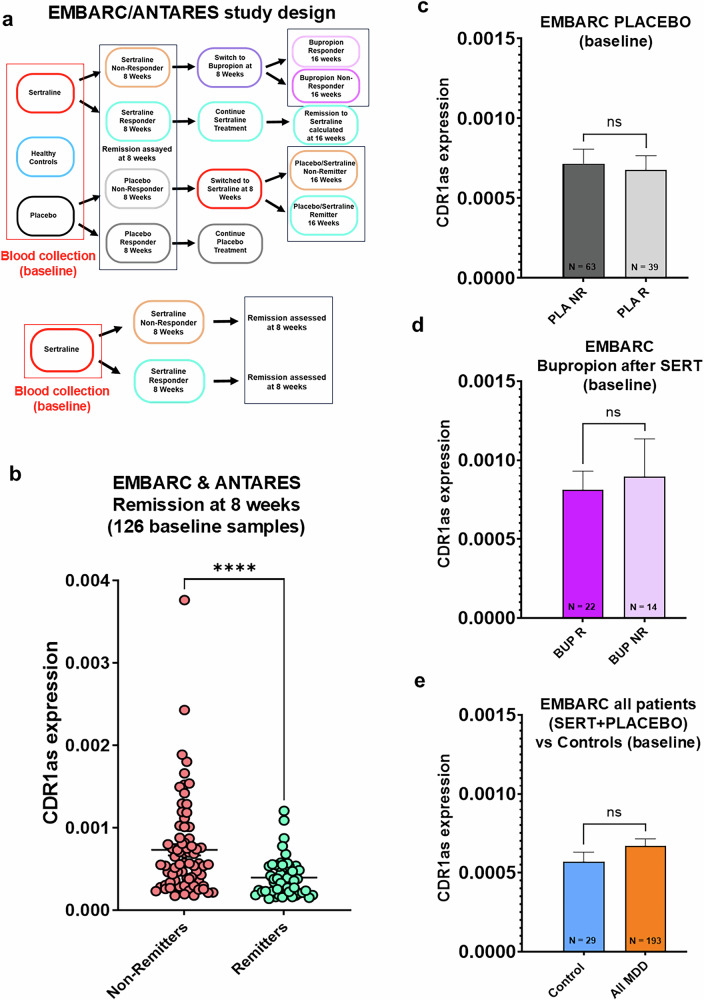
Blood CDR1as levels predict remission to sertraline but are not associated with response/remission to other treatments and MDD diagnosis. Schematic of the EMBARC and ANTARES antidepressant response studies (**a**). Rectangular shape indicates the study arms relative to the graphs included in this figure. Baseline whole blood CDR1as levels in the totality of the sertraline baseline samples from both clinical cohorts in remitters and non-remitters to sertraline treatment at 8 weeks (**b**). Baseline whole blood CDR1as expression levels CDR1as in Placebo responders (PLA-R) and non-responders (PLA-NR) (**c**). Baseline whole blood CDR1as expression levels CDR1as in Bupropion remitters (BUP-R) and non-remitters (BUP-NR) (**d**). Whole blood CDR1as baseline levels in healthy unaffected Controls and all MDD patients combined (**e**). For b-d, ****p < 0.0001, based on two-tailed Mann-Whitney test. Each graph is shown as Mean + SEM with the number of individual biological samples included within each graph.

We further assessed the biomarker potential of CDR1as on baseline samples from EMBARC MDD patients who were initially treated with Placebo, which due to lack of response were then switched to sertraline at 8weeks. Determination of response and remission after SERT treatment was then made at 16 weeks (i.e. 8 weeks after initiation of SERT treatment; see also Placebo arm of the study in Fig. 1b). Our results in these sertraline-treated patients of the Placebo arm of the study indicated that blood levels of CDR1as at baseline were significantly lower in patients who achieved response SERT at 16 weeks vs patients who did not (Fig. 1g), in agreement with our data in SERT-R and SERT-NR patients from the sertraline arm of the study. On a similar note, focused on remission at 8 weeks in both the EMBARC and ANTARES cohorts we observed a significantly higher expression in baseline CDR1as levels in non-remitters with sertraline vs remitters (Fig. 2a, b; 1.85-fold higher levels in SERT non-remitters). Looking at various demographics within the cohorts, such as age and sex, we did not observe any overall significant effects on CDR1as blood expression (Supplementary Table 2 and Supplementary Fig. 4a–c). It should be noted that the two studies employed different depression severity scales (MADRS for ANTARES; HAMD-17 for EMBARC). Moreover, although both studies recruited patients with moderate to severe depression, depression severity appeared to be modestly higher in ANTARES, when correcting for the differences between the MADRS and HAMD-17 [43] scales. Interestingly, differences in baseline CDR1as levels between SERT-R and SERT-NR were also significant in the subset of patients of African American/ Asian/ Pacific Islander race (Supplementary Fig. 4a), suggesting that the predictive value of this biomarker is consistent across the race groups examined in this study.

It is known that CDR1as functions as a sponge for miR-7a and protects it from degradation pathways involving the CYRANO lncRNA (also known as OIP5-AS1) [12, 30, 31, 33, 44]. However, unlike CDR1as, miR-7a and CYRANO do not exhibit an equally robust brain-enriched profile due to their expression in both brain and various peripheral tissues [45, 46]. Our results showed that CYRANO and miR-7a were not predictive of response to sertraline treatment and had no significant correlation with CDR1as in whole blood (Supplementary Fig. 5a–d). Furthermore, CYRANO expression was not enriched in the brain and miR-7a-5p exhibited a much less pronounced brain enrichment compared to that observed for CDR1as (Supplementary Fig. 5e–h). To determine the potential effects of comorbid anxiety on CDR1as expression, we compared whole blood CDR1as levels in patients with MDD with comorbid generalized anxiety disorder (GAD) as measured via the MINI diagnostic interview [36] in the ANTARES study vs MDD patients without GAD (Supplementary Fig. 6a). Our results showed no effect from comorbid GAD diagnosis in patients with MDD (Supplementary Fig. 6a). Furthermore, whole blood baseline CDR1as levels were significantly higher in SERT-NR vs SERT-R in the subset of ANTARES patients with MDD and either comorbid GAD or social anxiety (Supplementary Fig. 6b; using the MINI diagnostic interview [36]). On a similar note, no correlation between baseline CDR1as levels and the Holmes-Rahe Life Stress questionnaire score were observed in the ANTARES cohort (Supplementary Fig. 6c). In addition, no correlation was observed between baseline CDR1as expression and CRP protein levels in the same cohort (Supplementary Fig. 6d). We conclude that CDR1as is a brain-enriched circRNA specifically associated with prediction of response and remission with sertraline treatment in patients with MDD.

Focusing on the Placebo arm of the EMBARC study, we then measured CDR1as baseline levels in responders (PLA-R) and non-responders (PLA-NR) to 8 weeks of Placebo treatment using the same clinical criteria that were used for determination of response to sertraline (Fig. 2a). We observed no difference in baseline CDR1as levels in PLA-R and PLA-NR (Fig. 2c). To further test the specificity of CDR1as, we then looked at the Bupropion arm of the EMBARC study and we separated SERT-NR patients to those that achieved remission following a subsequent 8-week treatment with the atypical antidepressant Bupropion (BUP-R) and those that did not achieve remission following Bupropion treatment after failing to respond to sertraline (BUP-NR; see also Fig. 2a). Our results suggested that baseline levels of CDR1as were not indicative of remission following Bupropion treatment after failure to respond to sertraline (Fig. 2d). We then quantified the baseline expression of CDR1as in healthy unaffected Controls and compared them with all MDD patients. Our results indicated that CDR1as was not differentially expressed between MDD patients and Controls (Fig. 2e). Taken together, our results suggest that CDR1as is a reliable and specific biomarker associated with response to sertraline treatment.

### Blood CDR1as levels are responsive to sertraline treatment and are affected by family history of mania and diagnosis of bipolar disorder

**Fig. 3:**
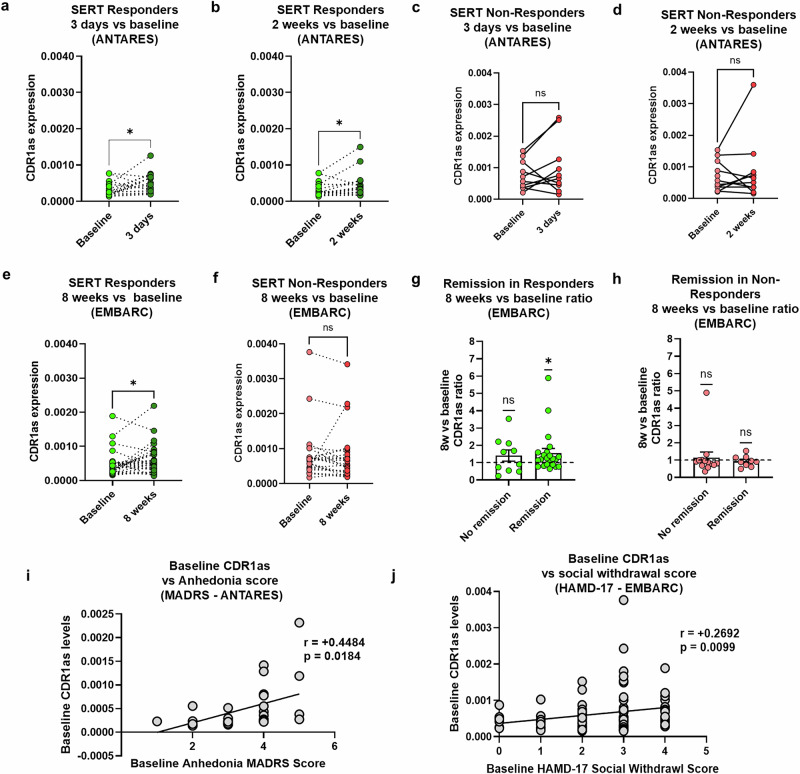
Longitudinal changes CDR1as expression levels are associated with remission with sertraline treatment. Whole blood CDR1as expression 3 days after sertraline treatment compared to baseline in SERT-R (**a**) of the ANTARES cohort. Whole blood CDR1as expression 2 weeks after sertraline treatment compared to baseline in SERT-R (**b**) of the ANTARES cohort. Whole blood CDR1as expression 3 days after sertraline treatment compared to baseline in SERT-NR (**c**) of the ANTARES cohort. Whole blood CDR1as expression 2 weeks after sertraline treatment compared to baseline in SERT-NR (**d**) of the ANTARES cohort. Whole blood CDR1as levels in baseline and 8 weeks of sertraline treatment in SERT-R (**e**) and SERT-NR (**f**). Ratio of 8 weeks vs baseline whole blood CDR1as levels in SERT-R patients who achieved or did not achieve remission with sertraline (**g**). Ratio of 8 weeks vs baseline whole blood CDR1as levels in SERT-NR patients who achieved or did not achieve remission with subsequent Bupropion treatment (**h**). Correlation between baseline CDR1as levels and baseline anhedonia MADRS scores in the ANTARES cohort (**i**). Correlation between baseline CDR1as levels and baseline social withdrawal scores in the HAMD-17 questionnaire of the EMBARC cohort (**j**). For a-f: *p < 0.05, based on two-tailed Wilcoxon matched-pairs signed rank test. For g-h, *p < 0.05, based on two-tailed one sample *t*-test (compared to mean ration of 1). Each graph from a-h is shown as Mean + SEM with individual biological samples values included within each graph. For i-j: Spearman correlation coefficient and two-tailed p-values are shown in the graphs.

Going back to the sertraline arm and taking advantage of the longitudinal nature of the EMBARC and ANTARES study sample collection, we measured the expression of CDR1as after 3days (ANTARES), 2 weeks (ANTARES) and, 8 weeks (EMBARC) of sertraline treatment and compared it to its levels at baseline within each patient. We found that levels of CDR1as displayed a significant increase after both 3 days (1.57-fold) and 2 weeks (1.41-fold) of sertraline treatment in the blood of SERT-R (Fig. 3a, b). However, levels of CDR1as in SERT-NR patients showed no significant increase at 3 days and were also unaltered compared to baseline levels at 2 weeks after treatment (Fig. 3c, d). At 8 weeks after treatment, levels of CDR1as were still significantly upregulated in SERT-R but not SERT-NR patients (Fig. 3e, f; EMBARC cohort). However, the increase at 8 weeks after treatment in SERT-R was reduced to only 1.25-fold above baseline levels (Fig. 3e), suggesting a gradual dampening of the post-treatment elevation of CDR1as with duration of treatment in responders to sertraline. Given that response to sertraline does not guarantee remission, we separated our SERT-R patients to remitters and non-remitters and compared the ratio of CDR1as levels at 8 weeks versus baseline for each patient for whom samples from both intervals were available. Our results indicated that SERT-R patients who achieved remission displayed a significant upregulation in CDR1as levels after 8 weeks of treatment (Fig. 3g). Importantly, no such changes were seen in SERT-R non-remitters (Fig. 3g), suggesting that CDR1as is responsive to treatment and displays a dynamic expression that can be used for disease monitoring and prediction of remission. Of note, no association was observed between changes in CDR1as levels at 8 weeks versus baseline and the possibility of remission in SERT-NR patients (Fig. 3h; patients receiving Bupropion treatment after failure to respond to sertraline). We conclude that CDR1as expression is affected by sertraline treatment and that the trajectory of changes in CDR1as levels post-treatment is associated with response and remission.

Looking at specific clinical dimensions with the strongest association with CDR1as expression within the MADRS and HAMD-17 depression severity scales, we found that anhedonia (as measured by the MADRS scale in ANTARES) and social withdrawal (as measured by the HAMD-34 scale in EMBARC) displayed the most notable positive correlation with baseline CDR1as expression in whole blood (Fig. 3i, j). Of note, whole blood CDR1as results in our laboratory in healthy controls were very stable in multiple measurements and were not affected by either fasting or eating breakfast or lunch or by the time of day that blood was collected Supplementary Fig. 7a–d). Moreover, the CDR1as PCR assay exhibited excellent accuracy and reproducibility with very low intra-run, inter-run, and lot-to-lot co-efficient of variation and appropriate linearity, dynamic range, and limit of detection (Supplementary Fig. 7b–f). Taken together, our results suggest that CDR1as is a stable blood biomarker associated with disease severity and specific MDD clinical manifestations.

### Accuracy of CDR1as blood assay for predicting response to sertraline

**Fig. 4:**
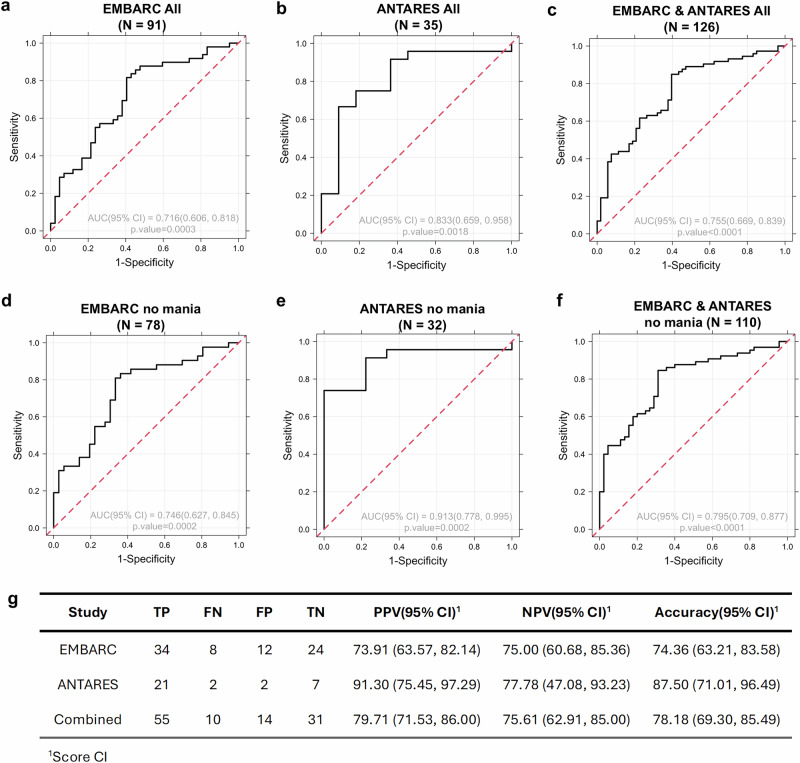
Accuracy of CDR1as blood assay for predicting response to sertraline. ROC analysis curve for CDR1as baseline levels between SERT-R and SERT-NR in the totality of the EMBARC (**a**) and ANTARES (**b**) cohort, as well as both cohorts together (**c**). ROC analysis curve for CDR1as baseline levels between SERT-R and SERT-NR after elimination of a total of 12 patients with positive family history of mania within the EMBARC (**d**) and ANTARES (**e**) cohorts, as well as both cohorts together (**f**). For (a-f) AUC (Area Under the Curve), p-values and confidence intervals are shown in the graphs. Table showing the PPV, NPV, and accuracy of the CDR1as assay in both cohorts, after elimination of patients with positive family history of mania (**g**). TP true positives, TN true negatives, FP false positives, FN false negatives.

To quantify the accuracy of CDR1as as a potential direct predictor of response assay for sertraline treatment, we performed a receiver operating characteristic (ROC) curve analysis on our baseline sertraline patient samples and calculated the area under the curve (AUC), as well as PPV, NPV, and corresponding accuracy of CDR1as as a single analyte (Fig. 4a–c). We found an AUC of 0.716 (p < 0.001) in the totality of the 91 baseline EMBARC samples examined (Fig. 4a) by utilizing a single cut-off for discriminating between SERT-R and SERT-NR (Ct = 30.7134; the Ct cutoff that achieved the best sensitivity and specificity in the first EMBARC discovery cohort data was selected and kept locked in all other datasets and analyses). We then kept the CDR1as Ct cutoff the same (30.7134) and repeated the AUC analysis in the ANTARES clinical cohort (N = 35). Our results showed a very good performance in this independent clinical validation cohort (Fig. 4b; AUC = 0.833, p = 0.0018). Combining both the ANTARES and EMBARC clinical cohorts we obtained an AUC of 0.755 (Fig. 4c; p < 0.0001). Taking into account the known heterogeneity of MDD, we hypothesized that confounding factors related to misdiagnosis or different subtypes of MDD could be affecting the performance of our assay. Since a small subset of patients initially diagnosed with MDD end up being later diagnosed with bipolar disorder [47, 48], we looked for potential clinical indicators of bipolar disorder susceptibility within our cohort. Given the hereditary risk of bipolar disorder [49], we focused on the presence or absence of family history of mania. Interestingly, we found that for the 12 patients who had a positive family history of mania (9 patients in the EMBARC cohort and 3 patients in the ANTARES cohort) there was no significant difference between SERT-R and SERT-NR mainly because of dampened CDR1as levels in the SERT-NR group (Supplementary Fig. 8a). To determine if the diagnosis of bipolar disorder could affect CDR1as expression we compared levels of whole blood CDR1as expression in adult patients with MDD and Bipolar Disorder in an additional naturalistic cohort from UTSW (RAD and Dallas-2K studies). Our results revealed a modest but significant downregulation in bipolar disorder vs MDD (Supplementary Fig. 8b). Given the above, we decided to re-analyze the performance of our assay on MDD patients that are negative for family history of mania to reduce the chances of comingling with patients with bipolar disorder that could be in the depressive phase of their symptoms. Our results showed an AUC of 0.746 for the EMBARC cohort without 9 patients with positive family history of mania (Fig. 4d; p = 0.0002), and an AUC of 0.913 in the ANTARES cohort without 3 patients with positive family history of mania (Fig. 4e; p = 0.0002). Looking at both cohorts together we achieved an AUC of 0.795 (Fig. 4f; p < 0.0001). Overall, our assay showed an accuracy of 78.2%, a PPV of 79.7% and an NPV of 75.6% (Fig. 4e). The sensitivity of the assay was 84.9% and the specificity at 68.9% (both cohorts included; the assay includes directions explaining the low performance in patients with family history of mania). These data suggest that CDR1as can be used as a single biomarker with acceptable accuracy for predicting response to sertraline treatment in MDD patients.

### SSRI class effect for CDR1as as a biomarker of antidepressant response

Although a reliable and accurate biological biomarker for predicting response to sertraline could result in significant benefits to the existing clinical workflow, a prediction of response to the whole class of SSRIs might provide physicians with additional flexibility for effective personalized treatment selection. On that end, we looked into the RAD and Dallas-2K naturalistic depression/bipolar disorder clinical studies at UTSW (Supplementary Fig. 9a). Despite the fact that there were no available baseline whole blood levels and HAMD-17 or MADRS scores in these studies to directly connect CDR1as expression to response and remission to SSRIs as done above for the EMBARC and the ANTARES cohort, there was availability of whole blood PAXgene sample collection and concomitant PHQ-9 scores at random intervals post-treatment for the majority of the samples (Supplementary Fig. 9a). Moreover, this cohort included different SSRIs, such as fluoxetine, citalopram, and escitalopram, in addition to sertraline, and included patients currently under treatment with SNRIs (duloxetine, venlafaxine, desvenlafaxine). We, therefore, focused on a subset of adult MDD patients under treatment with SSRIs (N = 17) that were faring well under treatment, defined as having a PHQ-9 score of 14 or lower. This set of 17 patients had an average PHQ9 of 6.1 and included 6 patients with Minimal Depression (PHQ-9: 0–4), 8 patients with Mild Depression (PHQ-9: 5–9), and 3 patients with moderate Depression (PHQ-9: 10–14). We then measured whole blood CDR1as expression in those 17 patients faring well under SSRI treatment as well as in 10 additional patients currently under SNRI treatment and determined based on the single cutoff of our assay if they would have been characterized as high likelihood to respond to SSRIs or low likelihood to respond to SSRIs based on CDR1as expression. Our results showed that levels of CDR1as even at different post-treatment intervals were able to correctly stratify the majority (70.6%) of adult MDD patients (12/17) faring well under SSRI treatment as high likelihood to respond to SSRI treatment with only 29.4% of these patients stratified as low likelihood to respond to SSRI treatment based on CDR1as expression (Supplementary Fig. 9b). On the other hand, 60% of adult patients under SNRI treatment exhibited CDR1as blood levels (at various intervals post-treatment) predictive of low likelihood to respond to SSRIs, with only 40% showing levels indicative of high likelihood to respond to SSRIs (Supplementary Fig. 9b; 10 for SNRI patients without stratification per PHQ-9). Regarding patients under SSRI treatment, the effect described above was not dependent on the type of SSRI treatment that was utilized (Supplementary Fig. 9c; no difference between the sertraline/fluoxetine group of adult patients (N = 4) and the escitalopram/citalopram group (13 adult patients). Moreover, looking at a small subset of adolescent MDD patients in this cohort faring well under SSRI treatment with the same PHQ-9 criteria used for the adult patients, we found 75% (3/4) being correctly stratified as high likelihood to respond to SSRI treatment based on CDR1as expression. Our results could suggest that blood CDR1as levels may correctly predict treatment outcomes for SSRIs as a class.

In order to further validate the predictive value of CDR1as in additional sample types, we utilized total RNA extracted from leukocytes collected from venous blood of patients with MDD treated with various classes of antidepressants as part of a naturalistic clinical study (NTC02209142; Supplementary Fig. 10a) [35]. Focusing on MDD patients who were prescribed various SSRIs and for which remission was calculated at 30 weeks after treatment, we found that CDR1as levels were significantly higher in non-remitters vs remitters (Supplementary Fig. 10b), and that despite the smaller size of this cohort, appeared to have a similar predictive effect (Supplementary Fig. 9c; AUC = 0.8750, p = 0.0550). Importantly, focusing on another subset of patients who were treated with other classes of antidepressants (SNRIs, TCAs, MAOIs), we observed no significant effects on CDR1as levels and remission following treatment (Supplementary Fig. 10d). Furthermore, levels of CDR1as in leukocytes from patients with MDD and Bipolar Disorder were not significantly different from unaffected healthy Controls (Supplementary Fig. 10e). Of note, expression of the brain-enriched CDR1as precursor long non-coding RNA LINC000632 (see also Supplementary Fig. 1a, c) was completely undetectable in either leukocytes from this cohort or whole blood-derived RNA from PAXgene (undetectable levels in 25 leukocyte and 25 whole blood RNA samples from patients and controls), further collaborating the outside of the blood source for CDR1as. We conclude that CDR1as levels might specifically predict treatment outcomes within the SSRI class of antidepressants, rather than solely sertraline.

### Levels of Cdr1as in mouse brain are regulated by serotonin receptor 5-HT2A activity

**Fig. 5:**
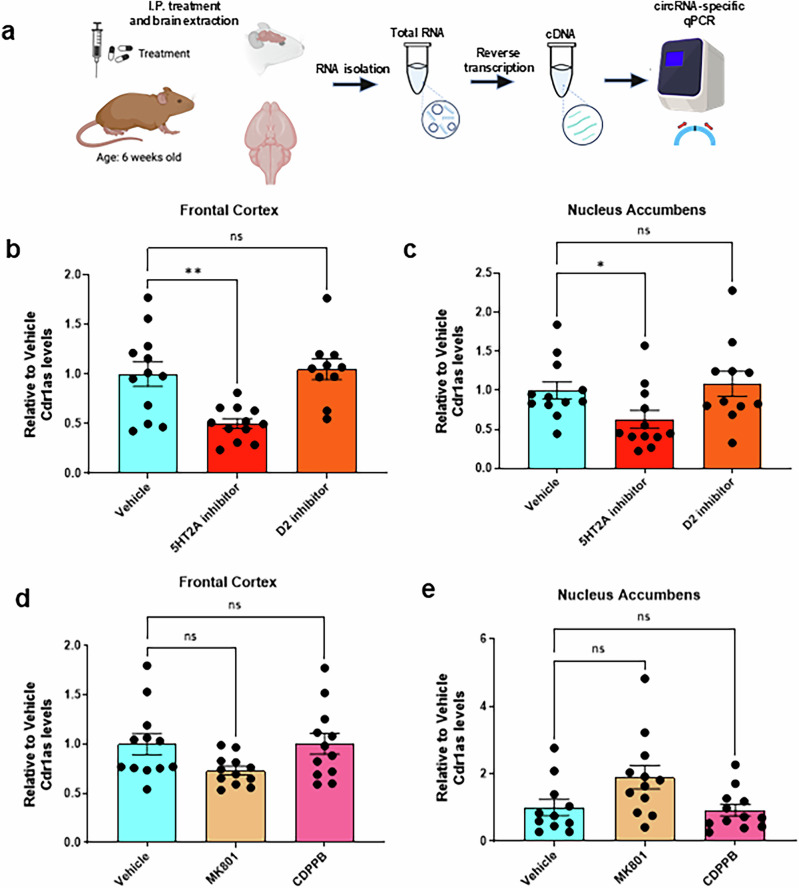
Cdr1as levels in mouse brain are inhibited following serotonin 5-HT2A receptor antagonism. Experimental design scheme (**a**). WT 5-week-old mice were treated with various pharmacological agents that aim the blockade of neuronal brain receptors. Mice were euthanized and different brain regions were dissected. Brain tissue from brain regions of interest was subjected to RNA extraction, reverse transcription, and circRNA qPCR. Mouse brain Cdr1as levels after treatment with a 5T2AR antagonist MDL100907 and the D2R antagonist sulpiride in mouse frontal cortex (**b**) and nucleus accumbens (**c**). Mouse brain Cdr1as levels after treatment with MK801, a selective NMDAR antagonist and CDPPB, an mGluR5 positive allosteric modulator in mouse frontal cortex (**d**) and nucleus accumbens (**e**). *p < 0.05, **p < 0.01, based on a one-way ANOVA with post-hoc Dunnett’s multiple comparisons test (b, d) or Kruskal Wallis ANOVA with Dunn’s multiple comparisons test (**c**, **e**). Each graph is shown as Mean + SEM with individual biological samples values included within each graph.

Even though a reliable molecular biomarker does not need to be necessarily associated with known disease-related molecular pathways, we decided to further investigate the potential mechanisms that could underlie the biogenesis of CDR1as within the brain. To that end, we took advantage of the fact that CDR1as is partially conserved between human and mouse and significantly enriched in mouse brain and neuronal cultures. We then treated mice with specific inhibitors targeting different neuronal receptors (glutamatergic NMDA receptor (MK801), D2 dopaminergic receptors (sulpiride), and 5-HT2A receptors (MDL100907). We also included treatment with the mGluR5 positive allosteric modulator, CDPPB. These receptors are important for brain function and cognition and relevant to the pathophysiology of depression (Fig. 5a) [50–54]. We found that serotonin 5-HT2A receptor but not D2 receptor antagonism was able to significantly downregulate Cdr1as in all three of the brain regions examined in this study (frontal cortex, nucleus accumbens, and putamen; Fig. 5b, c and Supplementary Fig. 11a). Furthermore, no effects were observed in mouse brain Cdr1as levels following either NMDA receptor antagonism or mGLUR5 receptor activation (Fig. 5d, e). Of note, focusing on another brain-enriched circRNA (circTulp4) [8, 10], we found no significant changes as a result of 5-HT2A receptor blockade, but an upregulation following D2 receptor antagonism in two for the three brain regions examined (Supplementary Figs. 11b–d). On the other hand, a significant downregulation in circTulp4 expression was observed in the nucleus accumbens of mice treated with MK801 (Supplementary Fig. 11e, f), suggesting that different brain-specific circRNAs are sensitive to diverse types of neuronal receptors. Our results suggest that Cdr1as expression in the mouse brain is regulated by serotonin receptor activity but is not significantly affected by glutamatergic NMDA/mGLUR5 and dopaminergic D2 receptor function.

### Neuronal expression of Cdr1as is regulated by BDNF receptor activity and involves the ERK/CREB signaling pathway

**Fig. 6:**
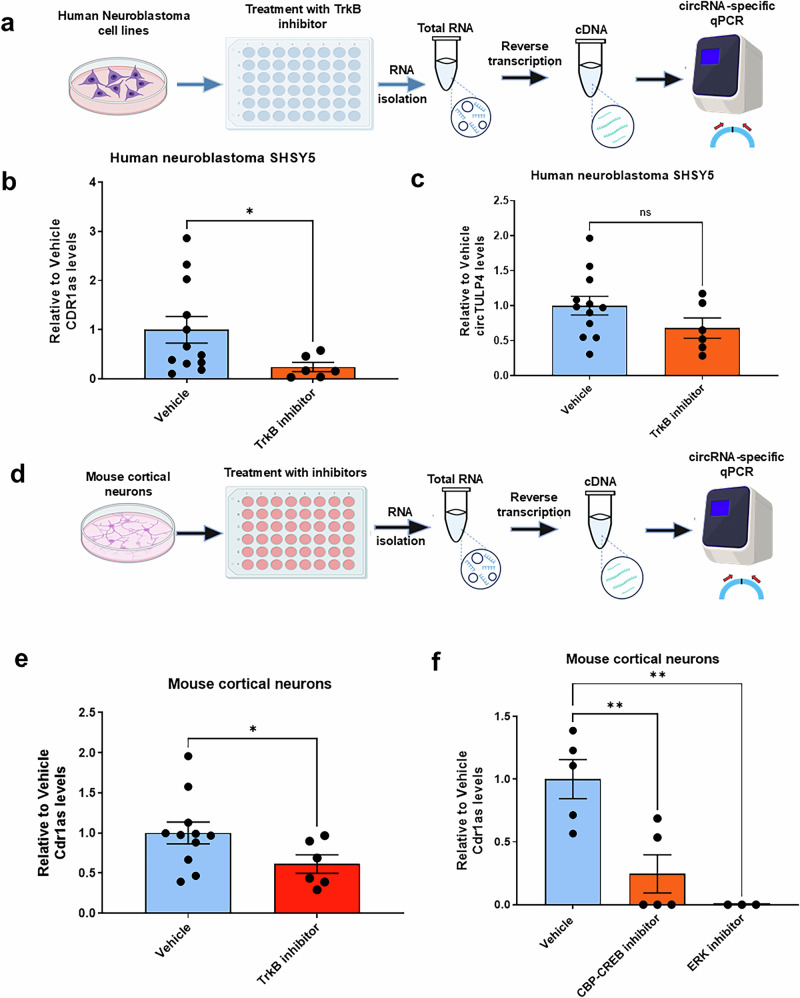
Neuronal CDR1as levels are influenced by BDNF receptor activity and ERK/CREB signaling. Schematic of the experimental design for the human neuroblastoma cell line (**a**). SHSY5 cells were cultured, plated in a well plate, and treated with various pharmacological inhibitors. At 24 h after treatment, cells were harvested, RNA was extracted, and reverse transcription and circRNA-specific PCR was performed. CDR1as (**b**) and circTulp4 (**c**) levels in human neuroblastoma SHSY5 cells after treatment with TrkB receptor antagonist. Experimental Design for mouse primary mouse cortical neuron experiments (**d**). Cdr1as levels in primary mouse cortical neurons after treatment with TrkB receptor antagonist (**e**). Cdr1as levels in primary mouse cortical neurons after treatment with CREB-CBP interaction inhibitor and a potent and selective ERK inhibitor (**f**). For (a) and (e): *p < 0.05 based on two-tailed (a) and one-tailed (e) Mann-Whitney test. For (f): **p < 0.01, based on a one-way ANOVA with post-hoc Dunnett’s multiple comparisons test. Each graph is shown as Mean + SEM with individual biological samples values included within each graph.

To further dissect the molecular pathways that could underlie the control of CDR1as expression within neurons, we utilized mouse cortical neuronal cultures and human neuroblastoma cell lines that were treated with different chemical inhibitors of molecular pathways associated with neuronal activity and known to play a role in depression and antidepressant action such as BDNF, as well as ERK and PKA signaling pathways [40, 55–57]. We initially tested the effects of BDNF receptor (TrkB) inhibition in human neuroblastoma SH-SY5Y cell lines, to determine if CDR1as could be downstream of neurotrophic cellular signaling (Fig. 6a). Our results showed that TrkB inhibition can significantly downregulate CDR1as expression in human neuroblastoma cells (Fig. 6b). This effect was specific, since levels of circTulp4 were not affected by TrkB inhibition (Fig. 6c). Importantly, repeating TrkB inhibitor treatment in mouse cortical neurons lead to a significant downregulation of Cdr1as expression (Fig. 6d, e). To further dissect the molecular pathways that could be associated with the control of neuronal CDR1as expression, we treated cortical neurons with CBP/CREB and ERK inhibitors. We found that Cdr1as was significantly downregulated by both treatments (Fig. 6e), suggesting that ERK/CREB signaling is upstream of Cdr1as synthesis. Additional treatments with PKA and PKC inhibitors showed no effects on circRNA expression in mouse cortical neurons (Supplementary Fig. 12a, b). Taking together all in vitro and in vivo experiments, we can surmise that Cdr1as expression in mouse neurons is activated via 5-HT2A- and BDNF/TrkB-mediated activation of ERK/CREB signaling (Supplementary Fig. 13).

## Discussion

Despite the progressive evolution in our understanding of the biological mechanisms associated with the pathogenesis of MDD [58, 59] and the further elucidation of molecular pathways with relevance to antidepressant response, there has been limited progress in the discovery of reliable and robust biological biomarkers with clinical relevance for MDD diagnosis and treatment. Here we provide data suggesting that baseline blood levels of a brain-enriched circRNA (CDR1as) associated with serotonin and BDNF receptor activity, can significantly predict response and remission following treatment with SSRI antidepressants. We further highlight the accuracy of a single analyte circRNA assay and demonstrate its specificity for the SSRI class of antidepressants. We provide additional mechanistic understandings of the molecular pathways that could regulate the expression of this circRNA in the CNS. Taken together, our data introduce a novel biological biomarker for predicting response to antidepressant treatment that is significantly associated with molecular cascades of relevance to antidepressant response within the brain.

Our data clearly demonstrate a robust enrichment in CDR1as and precursor CDR1as levels in human brain vs other human organs, which agrees with multiple previous studies [10, 18, 22, 30–33]. Furthermore, CDR1as is among the most readily-detected circRNAs in human cerebrospinal fluid, suggesting that it can cross the BBB [9, 60]. On a similar note, no synthesis of CDR1as precursor is detectable in whole blood and leukocytes, further validating that blood CDR1as expression is not due to local synthesis. Additional insights into the mechanisms of CDR1as secretion and trafficking are studies suggesting a significant enrichment of this circRNA in exosomes [61], which themselves are known to be able to cross the BBB [62]. The combination of particularly high levels of this circRNA in the human brain, its capacity to cross the BBB, and the very low expression of this circRNA in other human tissues, makes highly likely that levels of CDR1as in the blood are mostly derived from the brain. The fact that CDR1as levels in the blood are overall stable but appear to respond to sertraline treatment and our findings that 5-HT2A and BDNF receptor antagonism can reduce mouse brain Cdr1as levels further support this scenario.

Our EMBARC clinical study data suggest that whole blood levels of CDR1as are lower in SERT-R vs SERT-NR and that sertraline treatment leads to a significant upregulation in blood CDR1as levels only in SERT-R patients; an effect that is also associated with possibility for remission with sertraline. Of note, levels in unaffected Controls are similar to SERT-R levels, suggesting that physiological baseline levels of blood CDR1as are needed for response to sertraline and subsequent increase in such levels is required for remission with treatment. On the other hand, non-responders to sertraline show saturated levels in CDR1as expression that remain unchanged after treatment. Furthermore, the predictive value of CDR1as was limited to sertraline (EMBARC and ANTARES whole blood study) or the SSRI class of antidepressants (UTSW and naturalistic cohort and leukocyte clinical study) and not associated with response to placebo or other classes of antidepressants. Based on our animal and neuronal culture mechanistic data, CDR1as is downstream of serotonin and BDNF receptor signaling. Given that serotonin and BDNF signaling appear to be more closely linked to known effects of SSRI treatment within the brain, [50, 51] such findings suggest that CDR1as could be specifically linked to mechanisms of antidepressant response with relevance to the SSRI class of antidepressants. It is, thus, tempting, to hypothesize that the capacity to dynamically regulate CDR1as levels in brain and blood over a significant threshold and time period after treatment with SSRIs could provide a potential explanation for our findings. However, additional studies are required to further dissect the molecular mechanisms that could underlie the control of brain CDR1as expression and secretion into blood. Of note, baseline blood CDR1as levels are positively correlated with anhedonia and social withdrawal, both of which are a cluster of MDD symptoms that are strongly linked to MDD with melancholic features. Previous studies have suggested that patients suffering from depression with melancholic features are less likely to be prescribed SSRIs and potentially less likely to achieve response and remission following SSRI treatment [63, 64]. Additional work is therefore needed to determine whether a component of the prognostic potential of CDR1as is linked to different MDD endophenotypes.

Given the significant brain-enrichment of CDR1as, it is possible that disorders that significantly impact brain tissue or notably disrupt the BBB, such as traumatic brain injury or stroke, could confound blood CDR1as measurements. Since the clinical samples used in our study did not allow for such significant comorbidities, we are unable to fully characterize such effects, which is a limitation of our study. On a similar note, our results identified that the accuracy of our CDR1as assay is significantly improved if we exclude patients with family history of mania. Such findings suggest that reducing misdiagnosis between MDD and of other psychiatric disorders with depression symptomatology, such as bipolar disorder, could further increase the clinical efficacy of a molecular biomarker specifically predictive of SSRI response in MDD patients.

Previous studies have uncovered significant effects on synaptic gene expression, neuronal function and cognition following in vitro and in vivo manipulations of CDR1as and its downstream molecular targets [22, 33]. Specifically, it was shown that CDR1as KO mice display increases in immediately early gene expression due to deregulation in the expression of miR-7, a miRNA important for brain development and plasticity [65], for which CDR1as has more than 100 binding sites in its sequence [15, 22, 33]. Furthermore, loss of CDR1as expression led to significant elevations in excitatory neurotransmission, which impacted sensorimotor gating [22]. A recent study in neuronal cultures, further elucidated the interplay between CDR1as and miR-7 on glutamatergic neurotransmission and neuronal connectivity and presented evidence of CDR1as buffering and stabilizing miR-7 within neurons to influence neuronal synchrony and synaptic adaptation in an activity-dependent manner [33]. An additional study, found that synaptic expression of CDR1as in the medial prefrontal cortex was sensitive to fear extinction-training and that specific knockdown of CDR1as in neuronal processes of the infralimbic cortex affected fear extinction memory [32]. It is, thus, possible that CDR1as could be potentially found to be an important novel molecular component of the pathophysiology of antidepressant response. Future work is, thus, needed to further harness the power of this and other circRNAs for the better diagnosis and treatment of psychiatric and neurological disorders.

Our animal and neuronal culture studies identified neurotransmitter and neuromodulatory systems associated with depression as important for CDR1as biogenesis. Namely, we identified the 5-HT2A and BDNF/TrkB receptors as upstream regulators of CDR1as, since their inhibition resulted in reduced CDR1as expression. We also concluded after parsing a number of intracellular signal transduction cascades that the ERK/CREB molecular pathways also widely implicated in depression and antidepressant action [40, 56, 57] were important downstream regulators for neuronal CDR1as expression. Given that both 5-HT2A and TrkB receptor activation can result in ERK/CREB phosphorylation and activation, these results are consistent with our neuronal receptor data (see also Supplementary Fig. 13). Interestingly, both 5-HT2A and TrkB receptors were shown to enhance brain plasticity [66], while ERK/CREB activation has been associated with efficient and prolonged antidepressant response in a plasticity dependent-manner [67, 68]. It is, thus, tempting to hypothesize, that CDR1as expression in the brain and blood could be specifically controlled via the synergistic activation of serotonin and BDNF receptors. In such a hypothetical scenario, CDR1as levels in brain and brain-derived CDR1as levels in the blood could reflect the capacity of an individual patient to activate plasticity-dependent mechanisms following SSRI antidepressant treatment.

Our work, to the best of our knowledge, introduces the first ever brain-derived blood biomarker assay specifically associated with brain processes and molecular pathways relevant to depression and validated in multiple independent studies. Moreover, unlike the static nature DNA-based pharmacogenomics and their lack of clinical utility and efficacy in directly predicting antidepressant response, our circRNA biomarker has a dynamic nature via its responses to SSRI treatment and can directly predict response and remission to the SSRI class of antidepressants. Currently, without the guidance of our assay, only about 40% of patients with MDD respond to a first-line SSRI treatment. This suboptimal response rate and the current trial-and-error approach to antidepressant therapy mean that patients often endure an average wait of nearly a year to achieve satisfactory symptom management. This prolonged period imposes a significant burden on patients and their families and incurs substantial costs for insurers and the healthcare system. With a PPV of approximately 80%, our assay could significantly increase the percentage of patients responding to their initial treatment, fundamentally shifting clinical practice for depression. Furthermore, for the 60% of patients who currently do not respond to SSRIs as a first-line treatment, the existing process involves months of trying additional SSRIs before eventually switching to second and third lines of treatment. Given our assay’s NPV of close to 76%, it is anticipated that it could identify individuals unlikely to respond to SSRIs before treatment begins. This early identification would enable physicians to consider alternative treatment pathways at the earliest stages of a patient’s treatment journey. In summary, our assay has the potential to equip frontline healthcare providers with actionable guidance, substantially reducing the time to response and remission and lowering associated healthcare expenses. Future economic modeling and prospective economic utility studies are necessary to fully quantify the potential of this assay to revolutionize guided treatment for depression.

Our study introduces a tangible, brain-derived biological indicator of antidepressant treatment efficacy that utilizes a single circRNA biomarker specifically associated with predicting response to the SSRI class of antidepressants. By offering physicians information on the likelihood of response to antidepressant treatment, our assay could introduce a novel precision-medicine approach to guide treatment selection. Such an advancement, if expanded into other kinds of psychiatric drug treatments, could facilitate innovative mental health care approaches that are guided by science, optimized for each individual, and aimed at significantly alleviating suffering and ameliorating the quality of life of patients with MDD and other psychiatric conditions.

## Supplementary information

Supplementary Material

## Data Availability

The data that support the findings of this study are subject to intellectual property and proprietary information protection. Data are not publicly available but may be obtained from the corresponding author upon reasonable request and with permission from Circular Genomics Inc.
