# Correction: A brain-enriched circRNA blood biomarker can predict response to SSRI antidepressants

**DOI:** 10.1038/s41380-026-03579-3

**Published:** 2026-03-30

**Authors:** Grigorios Papageorgiou, El Chérif Ibrahim, Victor Gorgievski, Gabriella Maxson, Evelyn Lozano, Eric Gordon, Antoine Lefrere, Marie-Julie Toupet, Philippe Courtet, Raoul Belzeaux, Jane Foster, Thomas Carmody, Roy H. Perlis, Madhukar H. Trivedi, Eleni T. Tzavara, Nikolaos Mellios

**Affiliations:** 1 Circular Genomics Inc., San Diego, CA USA; 2 University of New Mexico, Department of Neurosciences, Albuquerque, NM USA; 3 Aix-Marseille Univ, CNRS, INT, Inst Neurosci Timone, Marseille, France; 4 Fondation FondaMental, Créteil, France; 5 Université Paris Cité, Inserm, CNRS, HealthFex, F-75006 Paris, France; 6 Hôpital Sainte Marguerite AP-HM, Pôle de Psychiatrie, Marseille, France; 7 IGF, Université de Montpellier, CNRS, INSERM, Montpellier, France; 8 Department of Emergency Psychiatry and Acute Care, Lapeyronie Hospital, CHU Montpellier, Montpellier, France; 9 Department of Psychiatry, CHU de Montpellier, Montpellier, France; 10 University of Texas Southwestern Medical Center, Department of Psychiatry, Dallas, TX USA; 11 Center for Quantitative Health, Massachusetts General Hospital, Boston, MA USA; 12 Department of Psychiatry, Harvard Medical School, Boston, MA USA

**Keywords:** Neuroscience, Genetics, Predictive markers

Correction to: *Molecular Psychiatry* 10.1038/s41380-026-03491-w, published online 17 February 2026

The originally submitted manuscript contained an error in the designation of the corresponding authors. Specifically, only one of the three co‑corresponding authors was marked with the corresponding‑author symbol and associated contact information. This presentation was incorrect and did not reflect the intended authorship structure.

This has now been corrected. The corresponding‑author symbol has been added to all three designated co‑corresponding authors, and the contact information for each author has been appropriately included in the revised manuscript to ensure accurate and complete attribution. The original article has been corrected.

