# Supplementary material for: A brain-enriched circRNA blood biomarker can predict response to SSRI antidepressants
Source: Mol Psychiatry. 2026 Feb 17;31(7):3637–52. doi: 10.1038/s41380-026-03491-w (PMC13269125; doi:10.1038/s41380-026-03491-w)
Supplement: Supplementary file 1 — Supplementary Material [file 41380_2026_3491_MOESM1_ESM.doc]

**A brain-enriched circRNA blood biomarker can predict response to SSRI antidepressants**

Grigorios Papageorgiou^1,2^, El Chérif Ibrahim^3,4^, Victor Gorgievski^5^, Gabriella Maxson^1^, Evelyn Lozano^1^, Eric Gordon^1^, Antoine Lefrere^4,6^, Marie-Julie Toupet^6^, Philippe Courtet^4,7,8^, Raoul Belzeaux^4,7,9^, Jane Foster^10^, Thomas Carmody^10^, Roy H Perlis^11,12^, Madhukar H. Trivedi^10#^, Eleni T. Tzavara^4,5,6#^, Nikolaos Mellios^1,2#*^.

1) Circular Genomics Inc., San Diego, CA. 2) Previously at: University of New Mexico, Department of Neurosciences, Albuquerque, NM. 3) Aix-Marseille Univ, CNRS, INT, Inst Neurosci Timone, Marseille, France. 4) Fondation FondaMental, Créteil, France. 5) Université Paris Cité, Inserm, CNRS, HealthFex, F-75006 Paris, France. 6) Hôpital Sainte Marguerite AP-HM, Pôle de Psychiatrie, Marseille, France. 7) IGF, Université de Montpellier, CNRS, INSERM, Montpellier, France. 8) Department of Emergency Psychiatry and Acute Care, Lapeyronie Hospital, CHU Montpellier, Montpellier, France. 9) Department of Psychiatry, CHU de Montpellier, Montpellier, France. 10) University of Texas Southwestern Medical Center, Department of Psychiatry, Dallas, Texas. 11) Center for Quantitative Health, Massachusetts General Hospital, Boston, MA. 12) Department of Psychiatry, Harvard Medical School, Boston, MA.

^*^ To whom correspondence should be addressed. [nmellios@circulargenomics.com](mailto:nmellios@circulargenomics.com)

^#^ Co-corresponding authors.

**Contents:**

**Pages 3-14: Supplementary Figures 1-14**

**Pages 15-19: Supplementary Figure legends**

**Page 20: Supplementary Table 1-2 and Table legends**

**Supplementary Figures**

**
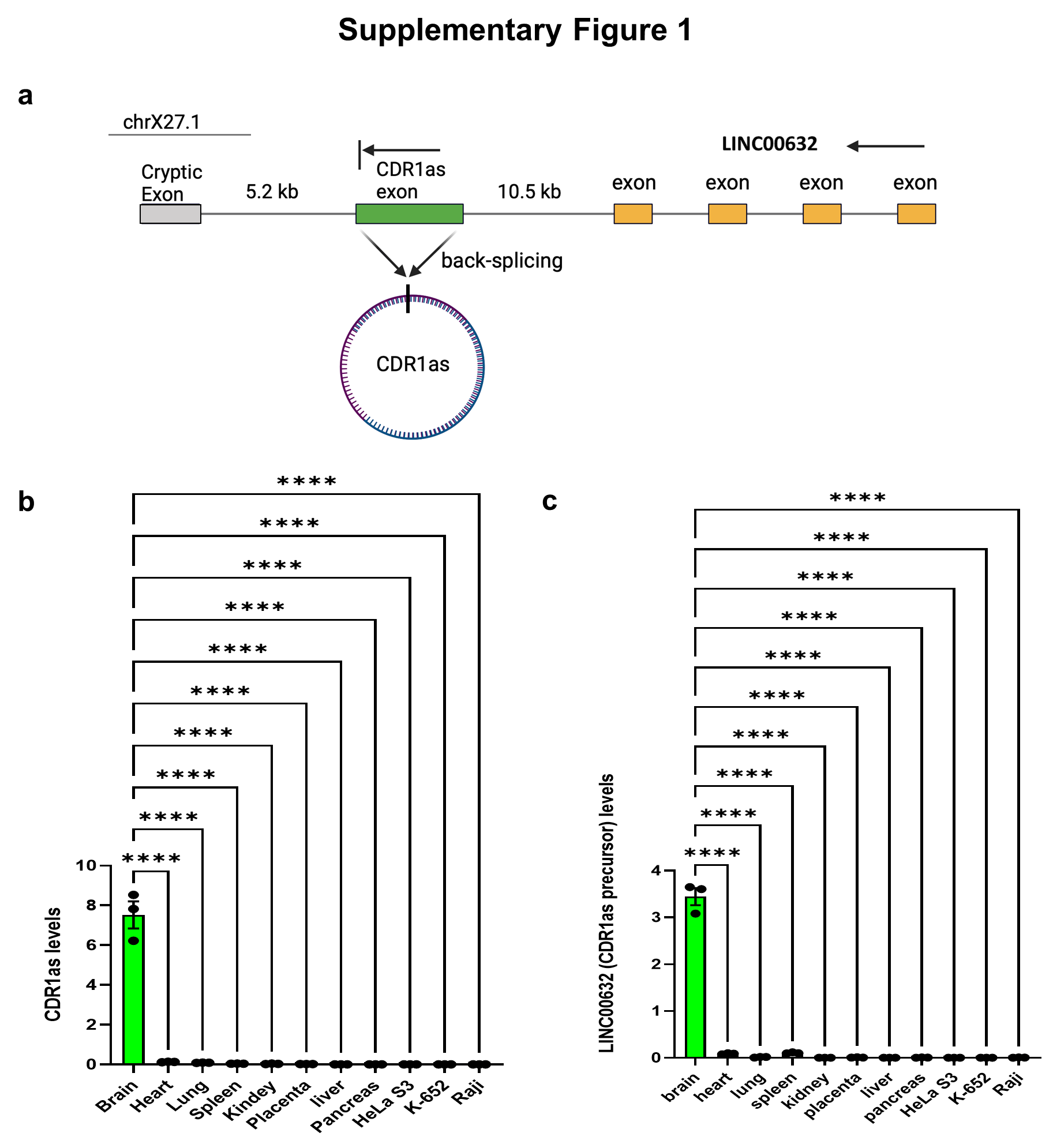
**


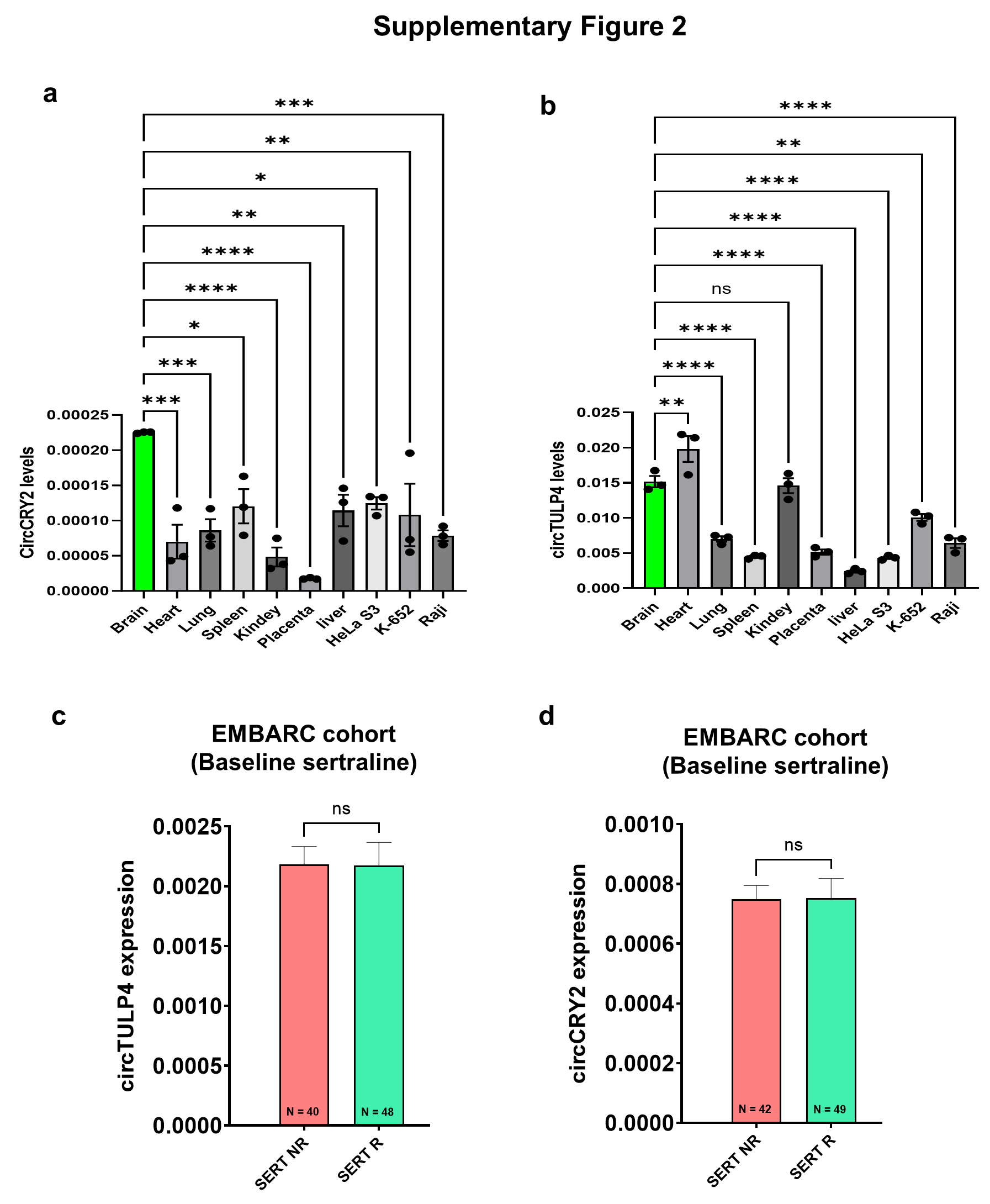


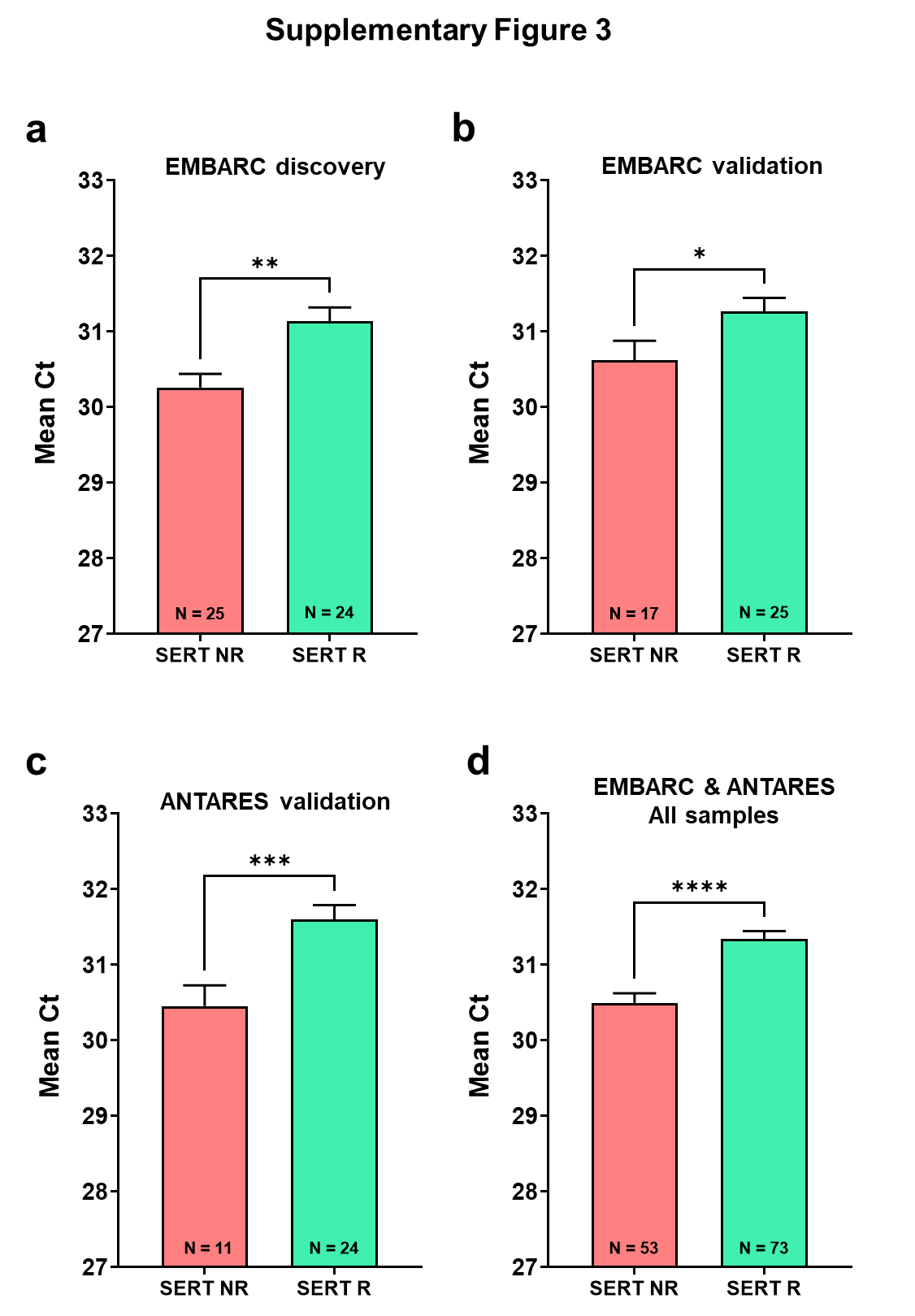


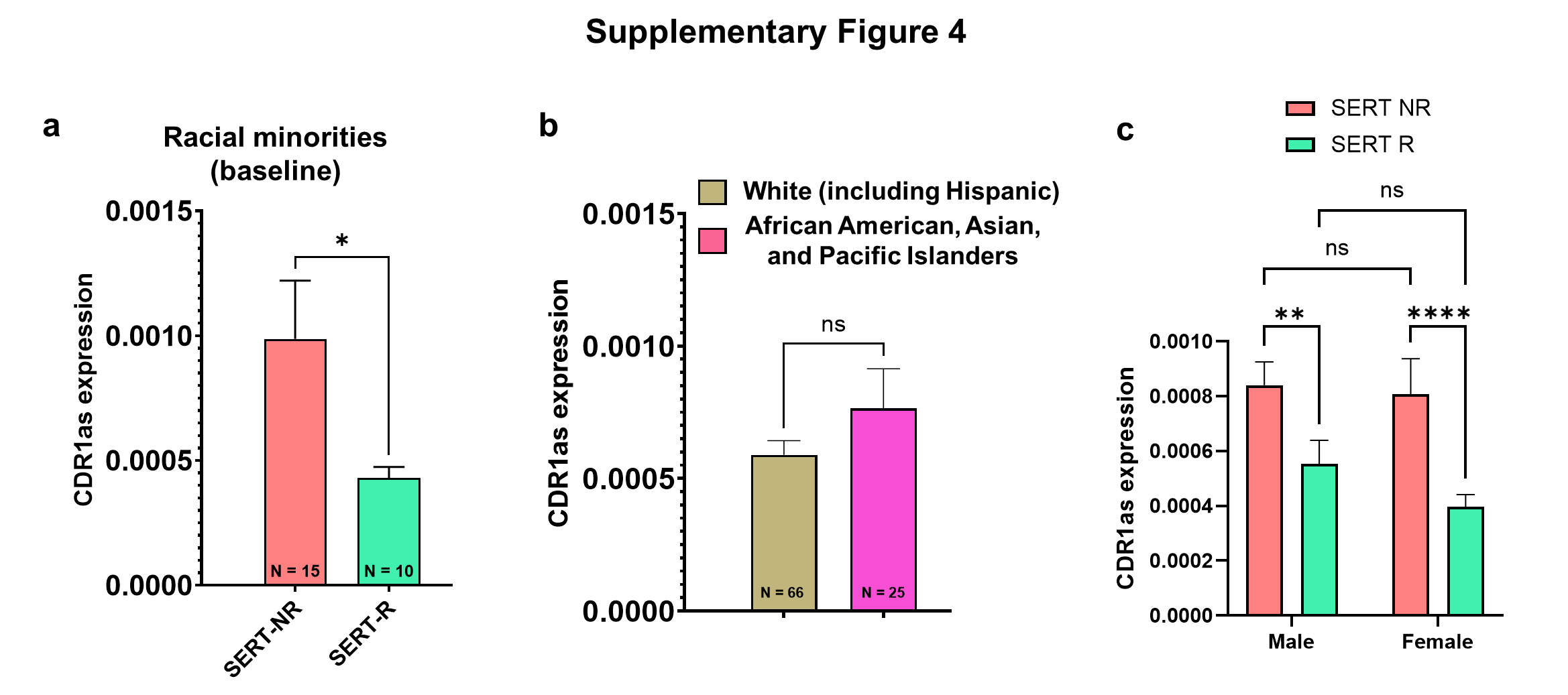


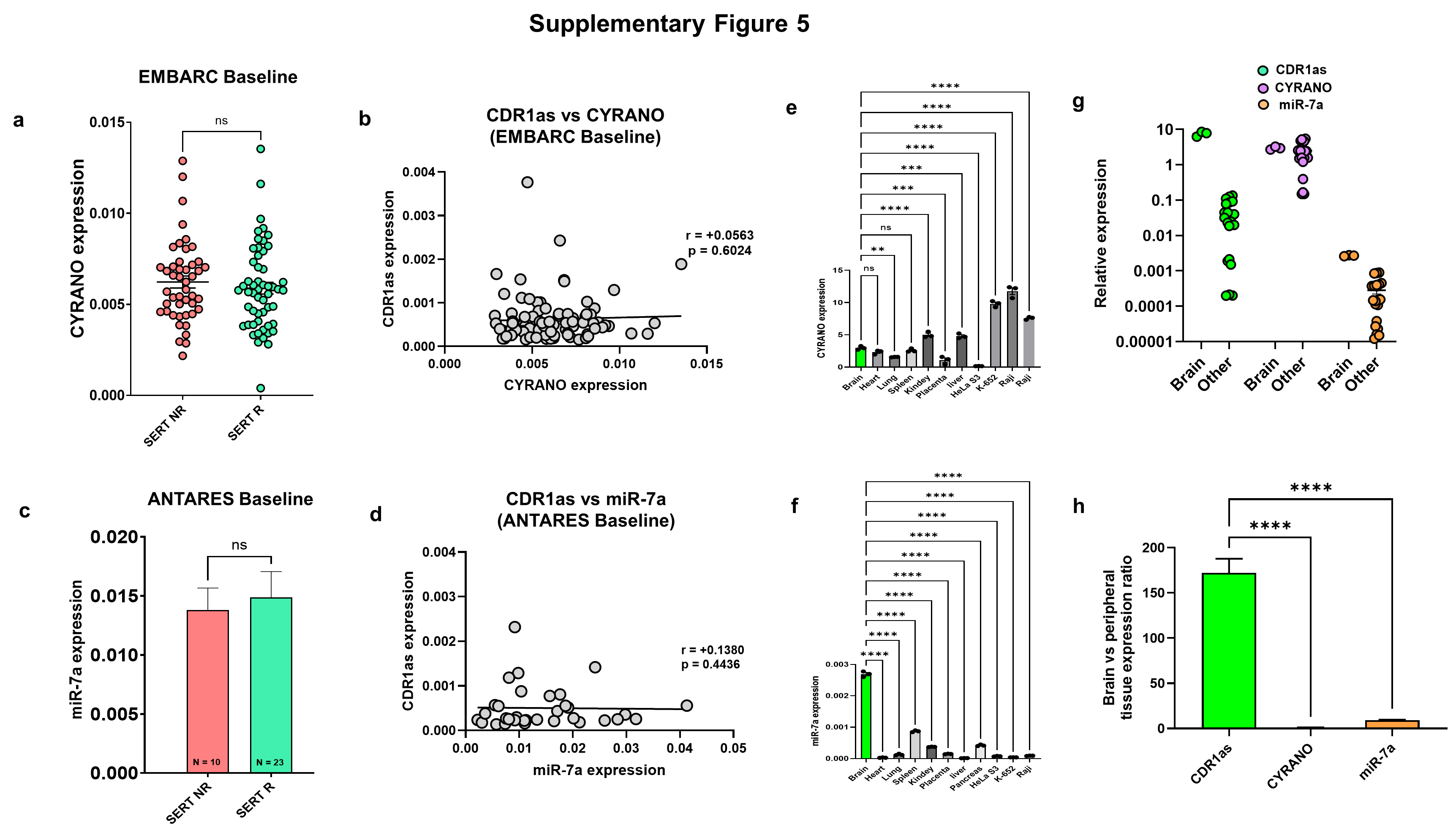


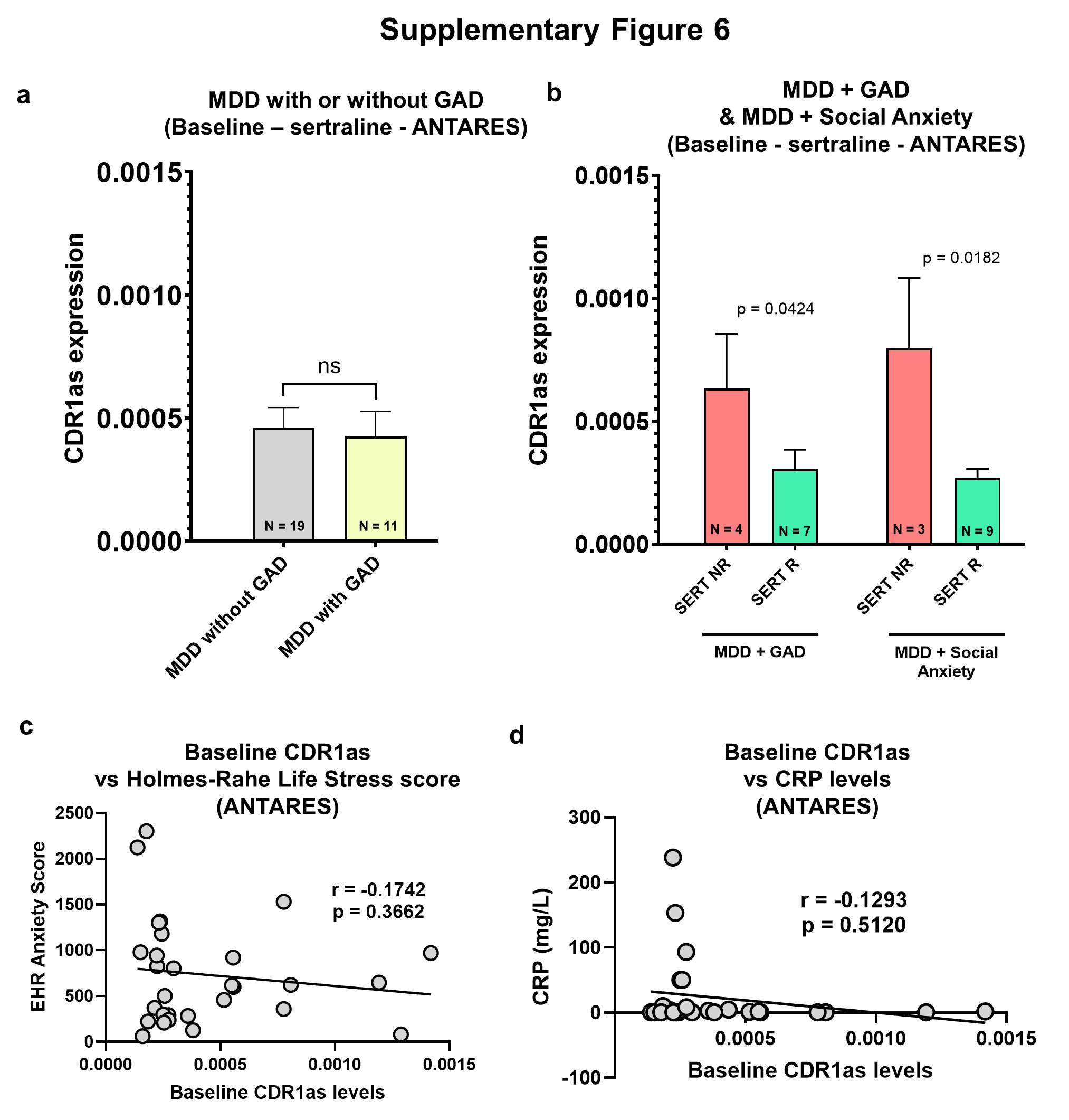


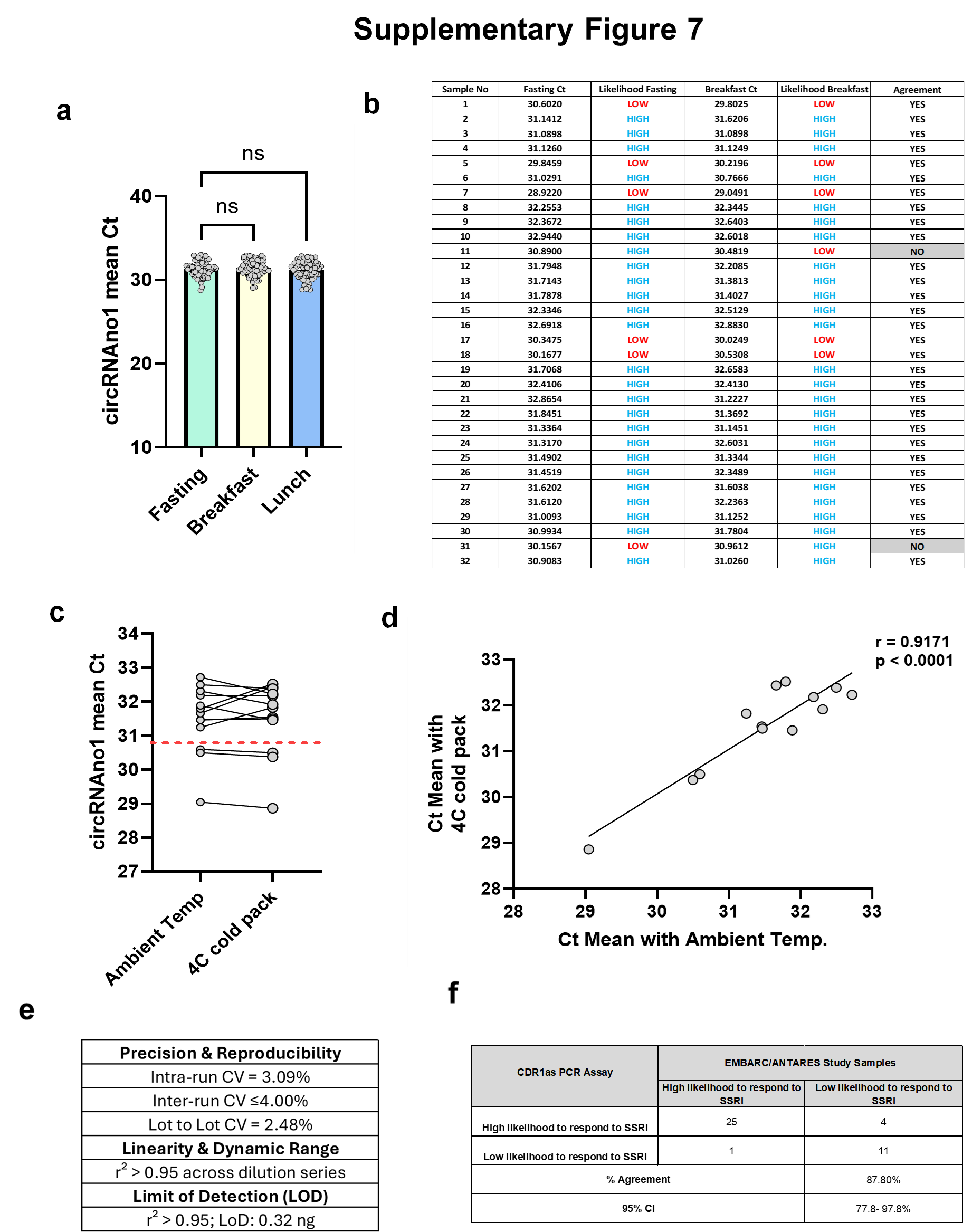


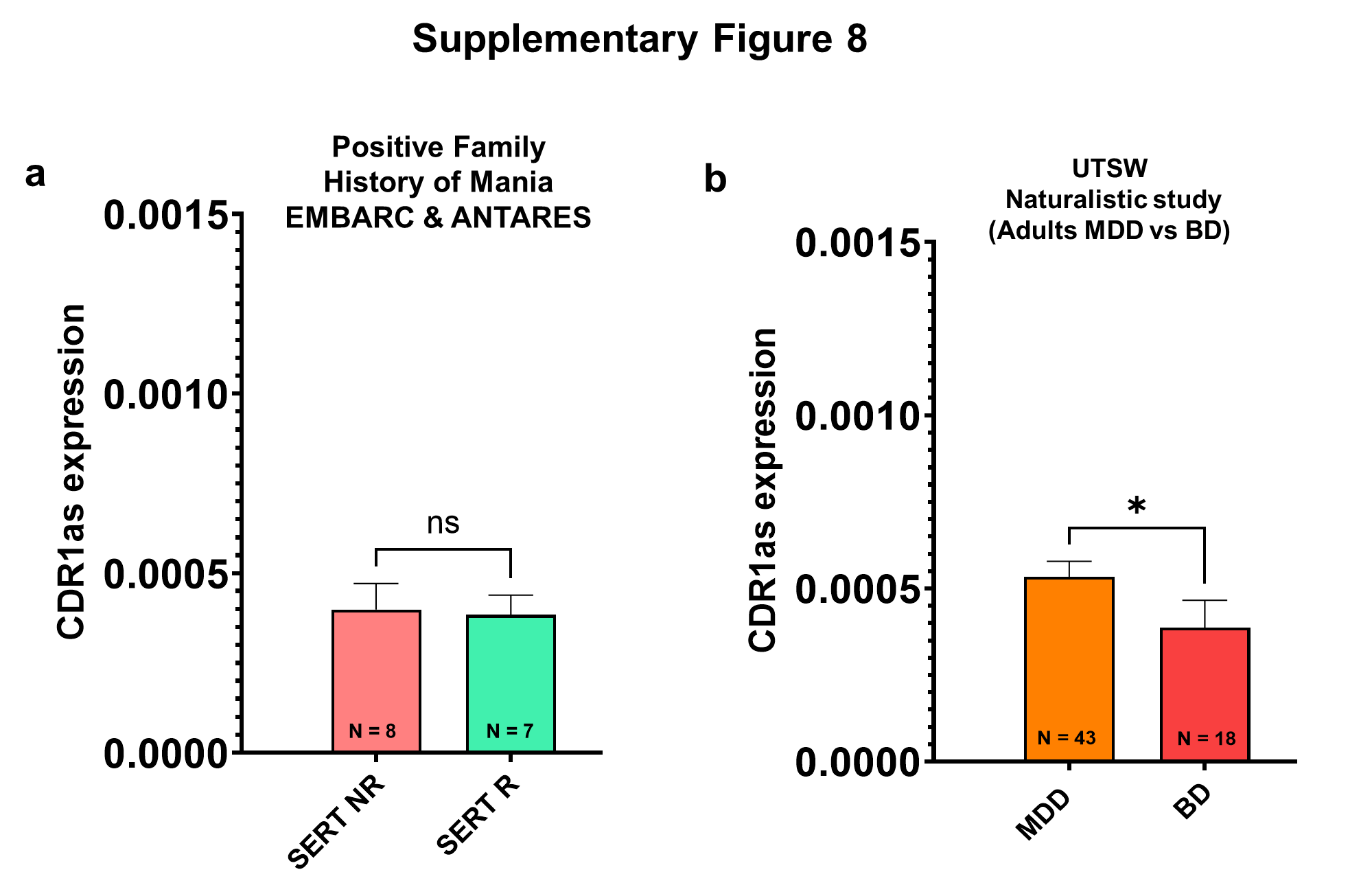


**
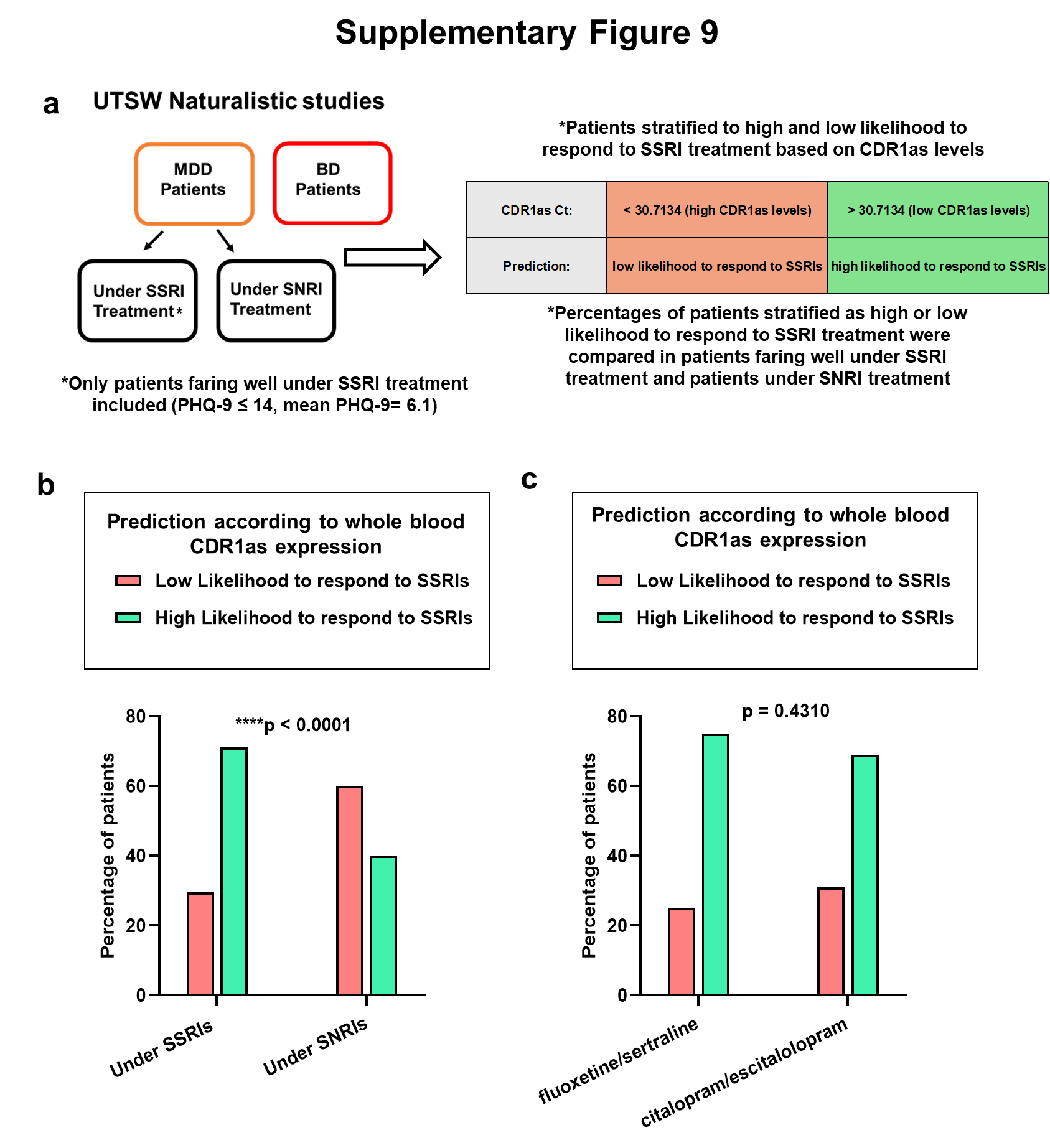
**

**
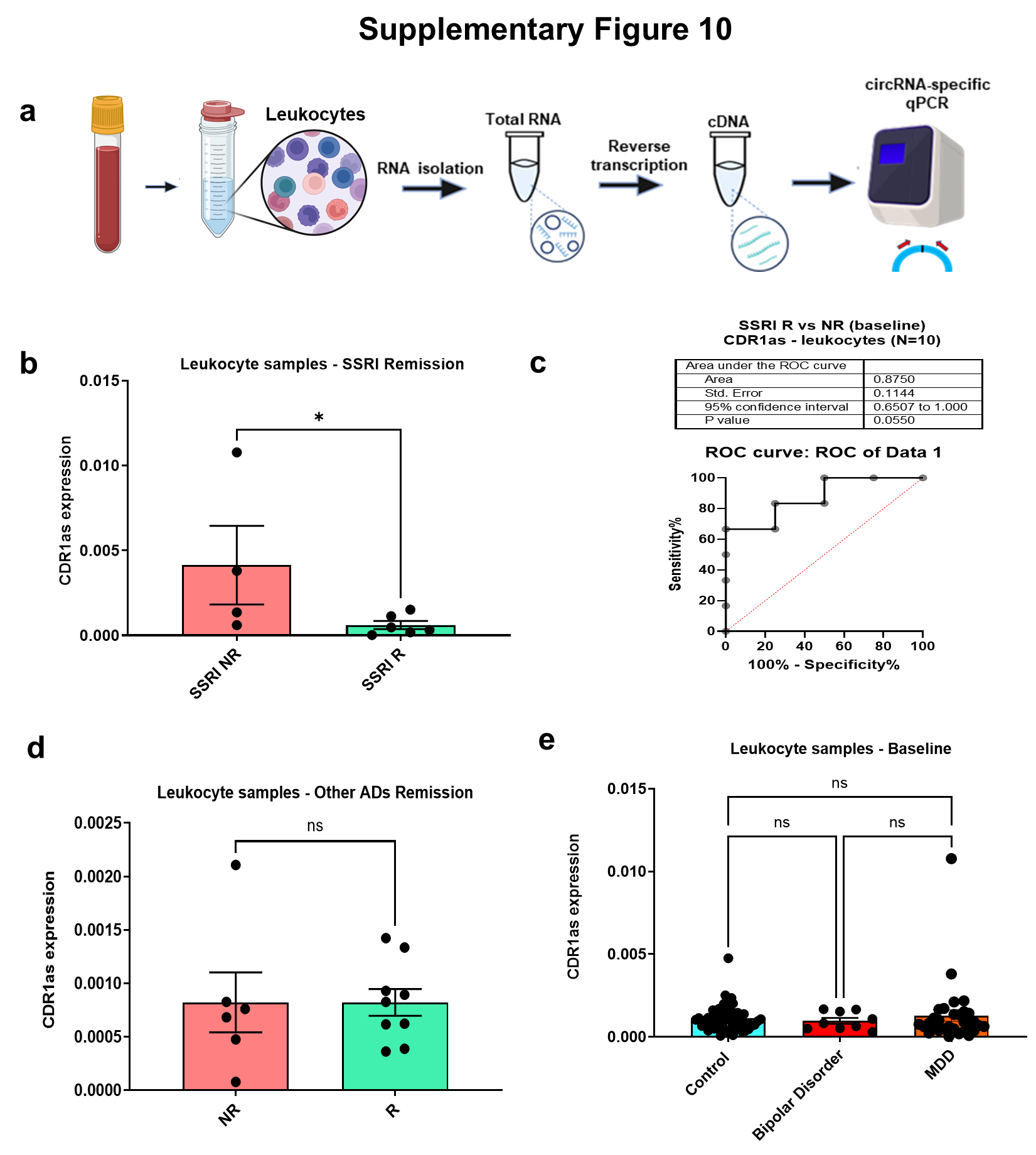
**

**
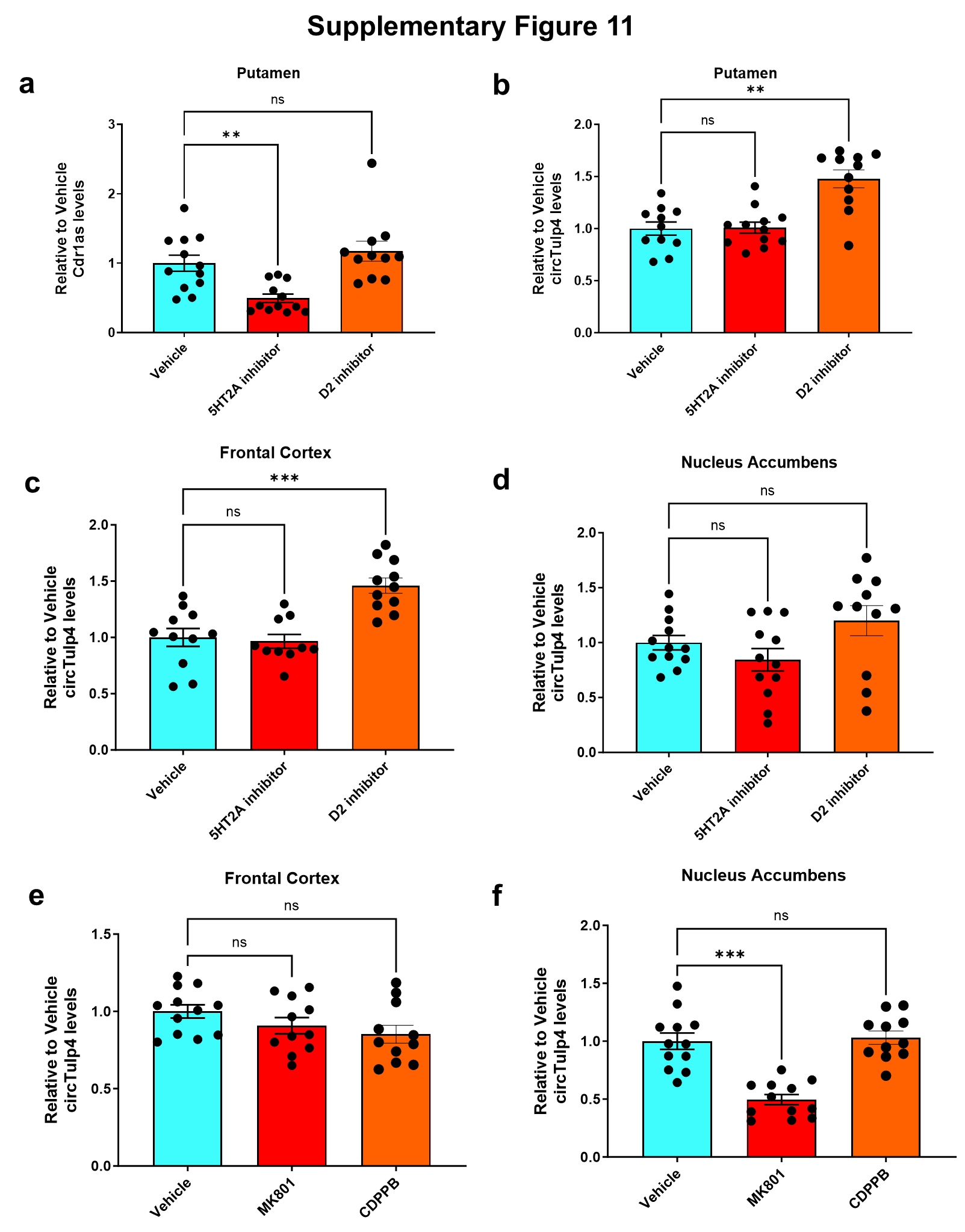
**

**
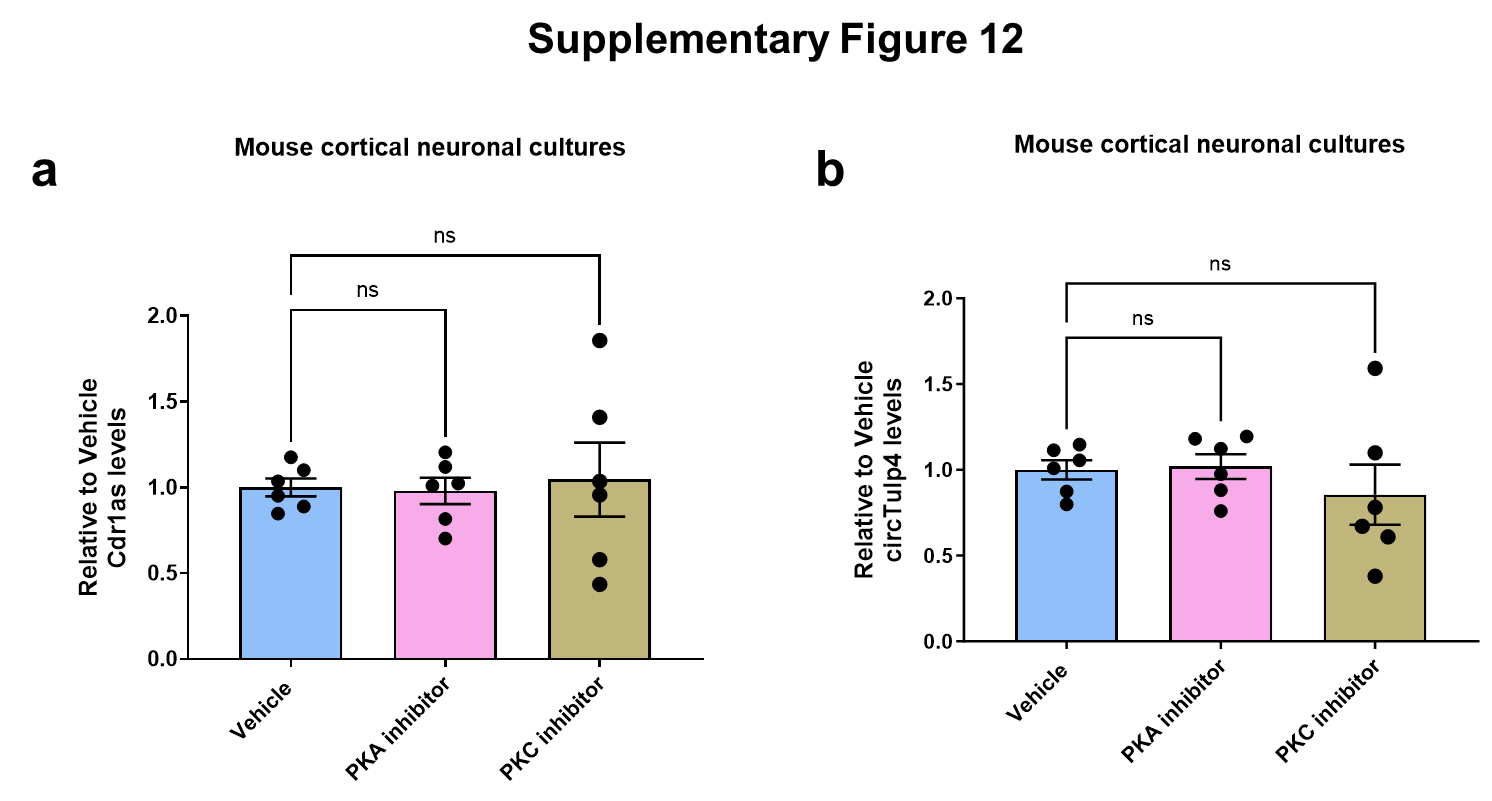
**

**
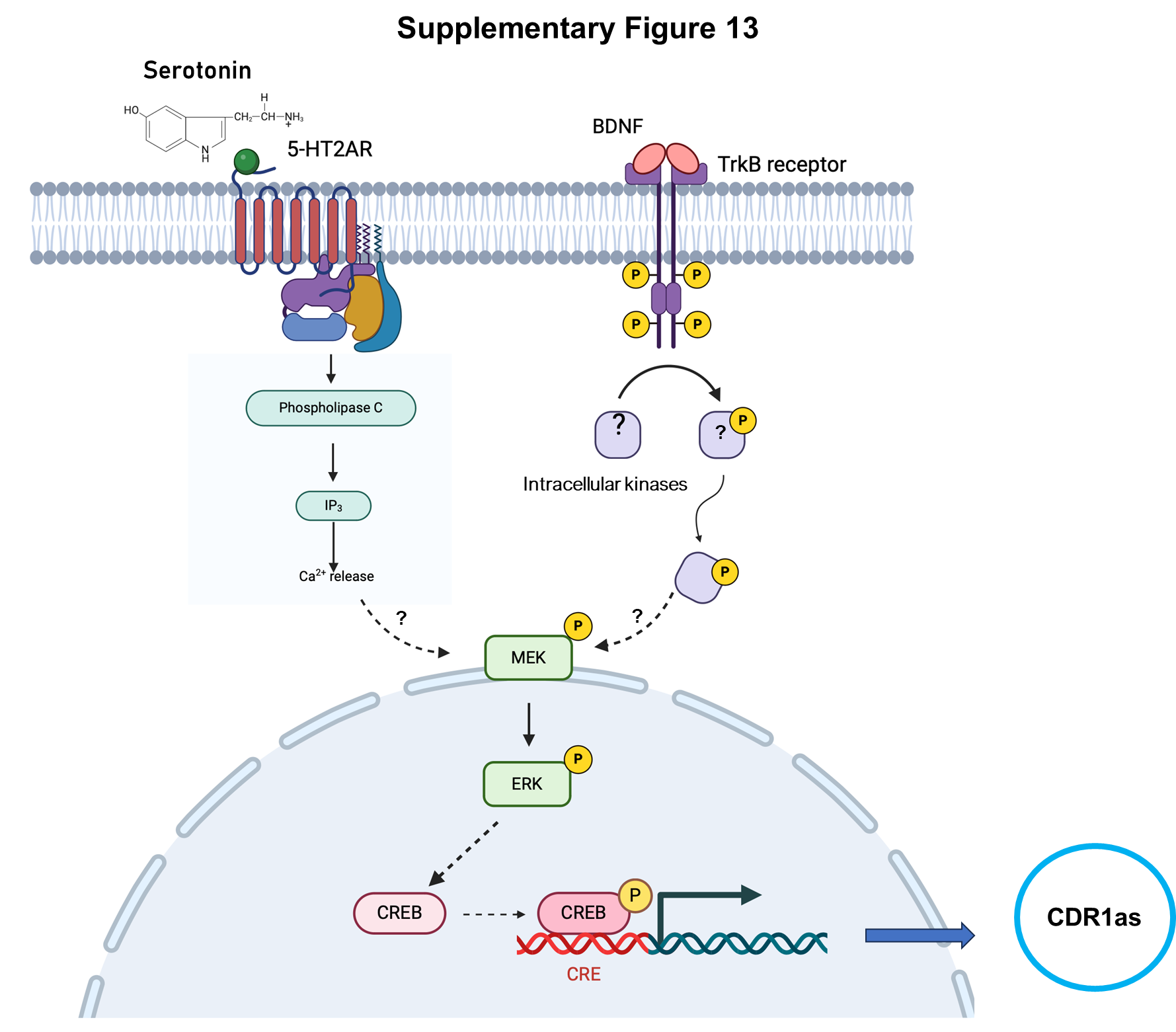
**

**Supplementary Figure Legends:**

**Supplementary Figure 1: CDR1as is particularly enriched in human brain.**

Schematic of the CDR1 antisense precursor RNA transcript (LINC000632). CDR1as is synthesized by the back-splicing and covalent joining of a single exon (exon 5; shown in green). Graph indicates CDR1as (**b**) and CDR1as precursor long non-coding RNA LINC00632 (**c**) levels based on circRNA-specific qPCR in total RNA from multiple human organs (brain, heart, lung, kidney, placenta liver, and pancreas) and cell lines (HelaS3, lymphoblast K-652 and Raji cells). The very highly expression of both CDR1asn and LINC000632 in the human brain tissue is clearly demonstrated. For (b) and (c): ****p < 0.0001, based on one-way ANOVA with post-hoc Dunnett's multiple comparisons test (vs Brain). Each graph is shown as Mean + SEM with individual biological samples values included as dots in (b,c).

**Supplementary Figure 2: Expression profile of circCRY2 and circTULP4.**

Graph indicates circCRY2 (**a**), and circTULP4 (**b**) levels based on circRNA-specific qPCR in total RNA from multiple human organs (brain, heart, lung, kidney, placenta, liver, and pancreas) and cell lines (HelaS3, lymphoblast K-652 and Raji cells). For (a) and (b): *p < 0.05, **p < 0.01, ***p < 0.001, ****p < 0.0001, based on one-way ANOVA with post-hoc Dunnett's multiple comparisons test (vs Brain). Baseline whole blood expression of circTULP4 in the EMBARC cohort Sertraline discovery cohort (**c**). Baseline whole blood expression of circCRY2 in the totality of the sertraline baseline samples from the EMBARC cohort (**d**). For (c-d), a two-tailed Mann-Whitney test was used. Each graph is shown as Mean + SEM with individual biological samples values included as dots in (a,b).

**Supplementary Figure 3: CDR1as Ct changes in EMBARC and ANTARES cohorts.**

Graphs showing the Mean + SEM Average Ct levels for baseline CDR1as in SERT-R and SERT-NR in the EMBARC discovery (a) and validation (b) cohorts (**a-b**). Graphs showing the Mean + SEM Average Ct levels for baseline CDR1as in SERT-R and SERT-NR in the ANTARES cohort (c) and in both cohorts (d) (**c-d**). For a and d: **p < 0.01, ****p < 0.0001, based on two-tailed Student’s *t*-test. For b-c: *p < 0.05, **p < 0.01, based on one-tailed Student’s *t*-test. Each graph is shown as Mean + SEM.

**Supplementary Figure 4: Effects of demographics on CDR1as expression**.

Baseline CDR1as levels in SERT-R and SERT-NR patients belonging to a racial minority (**a**; African American, Asian, and Pacific Islanders). Baseline CDR1as levels between white and racial minority patients (**b;** both SERT-R and SERT-NR included in each group). Baseline CDR1as levels in male and female SERT-R and SERT-NR patients from the combined EMBARC and ANTARES cohorts (**c**). Two-way ANOVA (sex x response status) showed no main effect for sex (F(1, 122) = 1.051; p = 0.3073, ns) and no significant interaction (F(1, 122) = 0.4598; p = 0.4990, ns). As expected there was a strong main effect of the response status (F(1, 122) = 14.38; p = 0.0002). **p < 0.01, and **** p < 0.0001, based on two-tailed Mann-Whitney test. Each graph is shown as Mean + SEM.

**Supplementary Figure 5: Baseline blood levels of CYRANO and miR-7a-5p do not predict response to sertraline treatment and relative tissue vs brain enrichment.**

Baseline whole blood expression of CYRANO lncRNA levels in baseline samples from the EMBARC cohort do not predict response to treatment (**a**). No correlation between CDR1as and CYRANO blood baseline levels in the EMBARC cohort (**b**). Baseline whole blood miR-7a-5p levels do not predict response to sertraline treatment in the ANTARES cohort (**c**). No correlation between CDR1as and miR-7a-5p blood baseline levels in the ANTARES cohort (**b**). For (a) and (c), p>0.05 (ns), based on two-tailed Mann-Whitney test. Each graph is shown as Mean + SEM. For (b) and (d), Spearman correlation coefficient and two-tailed p-values are shown in the graph. Graph indicates CYRANO (**e**), and miR-7a-5p (**f**) levels based on qPCR in total RNA from multiple human organs (brain, heart, lung, kidney, placenta, liver, and pancreas) and cell lines (HelaS3, lymphoblast K-652 and Raji cells). For (e) and (f): **p < 0.01, ***p < 0.001, ****p < 0.0001, based on one-way ANOVA with post-hoc Dunnett's multiple comparisons test (vs Brain). Relative brain and average of all other peripheral tissue expression for CDR1as, CYRANO, and miR-7a-5p (g). Relative to average peripheral tissue brain enrichment ratio for CDR1as, CYRANO, and miR-7a-5p (f). ****p < 0.0001, based on one-way ANOVA with post-hoc Dunnett's multiple comparisons test (vs CDR1as).

**Supplementary Figure 6: Anxiety and inflammation status do not affect CDR1as expression**.

Baseline whole blood levels of CDR1as do not differ between MDD patients with or or without comorbid generalized anxiety disorder (GAD) within the sertraline arm of the ANTARES cohost (**a**). Baseline whole blood levels of CDR1as predict response to sertraline treatment in the subset of MDD patients with comorbid generalized anxiety disorder (GAD) or social anxiety within the sertraline arm of the ANTARES study (**b**). No significant differences between baseline CDR1as whole blood levels and the Holmes-Rahe Life Stress questionnaire score (**c**). No significant correlation is observed between baseline CDR1as levels in the ANTARES cohort and baseline CRP protein expression (**d**). For (a-b), *p < 0.05, two-tailed Mann-Whitney test. Each graph is shown as Mean + SEM. For (c-d), Spearman correlation coefficient and two-tailed p-values are shown in the graph.

**Supplementary Figure 7: Reproducibility and analytical properties of CDR1as PCR assay.**

Average Ct values for whole blood CDR1as expression remain unaffected by time of day and fasting or lack thereof (**a**). Whole blood samples from 40 healthy Controls were collected either early in the morning after overnight fasting (fasting group), later in the morning after having breakfast (breakfast group; N = 68), or in the afternoon after having lunch (lunch group; N = 64) to test for effects of fasting/eating and time of the day (N = 70). ANOVA with Dunnett's multiple comparisons test was used. For (a), *p < 0.05, based on two-tailed Mann-Whitney test. Each graph is shown as Mean + SEM. Table showing circRNAno1 mean Ct values and likelihoods to respond to SSRI comparisons for 32 individuals for which PAXgene blood was collected in the morning after either overnight fasting (Fasting) or breakfast earlier in the morning (Breakfast) (**b**). Notice agreement on likelihood to respond predictions in 30 out of 32 cases. CircRNAno1 mean Ct values for 13 individuals with shipment of PAXgene tubes in either ambient temperature packing (Ambient Temp) or 4C cold packing (4C cold pack) (**c**). Notice a 100% agreement on Ct means been above or below the single Ct cutoff of 30.7134. Correlation between the mean Ct values shown in figure 2c showing a very high correlation in Ct values between the two shipment methods (**d**, Pearson r coefficient and two-tailed p-value are shown in the graph). Summary of analytical validation parameters for the CDR1as PCR assay (**e**, **f**).

**Figure 8: Effects of family history of mania and BD diagnosis on CDR1as expression.**

Baseline whole blood CDR1as levels in patients with family history of mania do not show significant prediction of response to sertraline treatment (**a;** results from both EMBARC and ANTARES cohort combined). Baseline whole blood CDR1as levels are significantly downregulated in patients with diagnosis of BD vs MDD (**b**). For both graphs, *p < 0.05, based on two-tailed Mann-Whitney test. Each graph is shown as Mean + SEM.

**Supplementary Figure 9: CDR1as levels correctly stratify patients faring well under SSRI treatment vs patients on SNRI treatment.**

Schematic of the UTSW naturalistic study and explanation of experimental procedure to use whole blood CDR1as levels (at various intervals after treatment) to stratify patients as high or low likelihood to respond to SSRIs and utilize concomitant PHQ-9 scores to identify the performance of such predictions in adult MDD patients faring well under SSRI treatment vs adult MDD patients currently on SNRIs (**a**). The majority (70.6%) of adult MDD patients (12/17) faring well (defined as having PHQ-9 score of 14 or lower; average PHQ9 for these 17 patients was 6.1) under SSRI treatment were correctly predicted by CDR1as levels (at various intervals post-treatment) to be high likelihood to respond to SSRI treatment vs 29.4% that were predicted to be low likelihood to respond in the UTSW naturalistic study (**b**). On the other hand, 60% of adult patients under SNRI treatment exhibited CDR1as blood levels (at various intervals post-treatment) predictive of low likelihood to respond to SSRIs, with only 40% showing levels indicative of high likelihood to respond to SSRIs (**b**; total N = 10 for SNRI patients without information on PHQ-9). This effect was not significantly influenced by the type of SSRI treatment that was utilized (**c**; no difference between the escitalopram/citalopram group of 13 patients and the fluoxetine/sertraline group of 4 patients. For (e-f) Chi-square with Yates' correction was used with p-values shown in the graphs.

**Supplementary Figure 10: CDR1as levels in human leukocytes can specifically predict remission following treatment with SSRIs**.

Schematic of the experimental design (**a**). Leukocytes were isolated from human whole blood as described in Materials and Methods and CDR1as expression was quantified with qPCR. Baseline leukocyte CDR1as expression levels in patients who either achieved remission (SSRI-R) or not (SSRI-NR) following 30 weeks of SSRI treatment (**b**). ROC analysis curve for baseline CDR1as levels in leukocytes between SSRI remitters and non-remitters (**c**). The AUC (Area Under the Curve) along with the table of statistics is included. (D Baseline leukocyte CDR1as expression levels in patients who either achieved remission (R) or not (NR) with other classes of antidepressants (Ads) following 30 weeks of treatment (**d**). ADs included SNRIs, TCAs and MAOIs. Baseline CDR1as levels in leukocytes from patients with MDD compared to Bipolar Disorder and unaffected healthy Controls (**e**). For (b): *p < 0.05, based on one sample t-test. One-way ANOVA with Tukey's multiple comparisons test was used in (d).

**Supplementary Figure 11: Expression levels of circTulp4 across various mouse brain regions after different *in vivo* pharmacological experiments and Cdr1as levels in putamen following 5-HT2A and D2 receptor inhibition.**

Mouse brain Cdr1as (**a**) and circTulp4 (**b**) levels after treatment with a pure 5T2AR antagonist MDL100907 and the D2R antagonist Sulpiride in mouse putamen. Mouse brain circTulp4 levels after treatment with the 5T2AR antagonist MDL100907 and the D2R antagonist Sulpiride in mouse frontal cortex (**c**) and nucleus accumbens (**d**). Mouse brain circTulp4 levels after treatment with MK801, a selective NMDAR antagonist and CDPPB, an mGluR5 positive allosteric modulator in mouse frontal cortex (**e**) and nucleus accumbens (**f**). **p < 0.01, ***p < 0.001, based on one-way ANOVA with post-hoc Dunnett's multiple comparisons test (c, e) or Kruskal Wallis ANOVA with Dunn's multiple comparisons test (a, b). Each graph is shown as Mean + SEM with individual biological samples values included within each graph as dots.

**Supplementary Figure 12: Cdr1as and circTulp4 mouse levels in cortical neurons following *in vitro* PKA and PKC inhibitor treatments.**

Primary mouse cortical neuron Cdr1as (**a**) and circTulp4 (**b**) levels, based on qPCR, and after treatment with PKA and PKC (small molecule kinase inhibitors). One-way ANOVA with post-hoc Dunnett's multiple comparisons test was used for both (a) and (b). Each graph is shown as Mean + SEM with individual biological samples values included within each graph as dots.

**Supplementary Figure 13: Proposed schematic of CDR1as regulation within neurons**

5-HT2A receptors are found on the postsynaptic membrane of neurons and are G-protein coupled receptors that can activate (among others) the PLC- IP3 pathway, thus leading to the release of Ca+2 from the Endoplasmic Reticulum (ER). This elevation of Ca+2 levels can activate the downstream protein kinase MEK, which in turn will phosphorylate ERK1/2, resulting in activation of CREB. Tropomyosin receptor kinase B (TrKB) is a transmembrane receptor protein that binds to Brain-derived neurotrophic factor (BDNF). Upon activation of this receptor, autophosphorylation can occur with activation of a series of downstream intracellular kinases (such as Ras-Raf, Akt, PI3K), which can also result in elevation of intracellular Ca+^2^ levels. In a common pathway to 5-HT2A receptor activation, such increases in Ca+^2^ levels can phosphorylate MEK and ERK1/2, thus leading to further CREB activation. CREB-mediated transcriptional effects of yet unknown gene targets then result in the synthesis of CDR1as within the brain.

**Supplementary Table 1 -** Primer information. Table showing the primer name, species, transcript, and assay ID or primer sequence.

| **Primer name** | **Species** | **Transcript name** | **Primer Sequence or Assay ID** |
| --- | --- | --- | --- |
| h_CDR1as-F | Human | CDR1as | ACGTCTCCAGTGTGCTGA |
| h_CDR1as-R | Human | CDR1as | CTTGACACAGGTGCCATC |
| h_circCRY2-F | Human | circCRY2 | GAGGAACCACCTGGTGAAGA |
| h_circCRY2-R | Human | circCRY2 | TTCAGTGGGGAACCTCTCAG |
| h_circTULP4-F | Human | circTULP4 | GGAGTGGTTGGGGTGACTTT |
| h_circTULP4-R | Human | circTULP4 | TCAACTGCCATACGAAGCGT |
| h_LINC000632_Taqman | Human | LINC000632 | Hs03988162_m1 |
| h_CYRANO_Taqman | Human | CYRANO (OIP5-AS1) | Hs03677189_g1 |
| m_Cdr1as-F | Mouse | Cdr1as | GTGTCTGCCGTATCCAGGGTT |
| m_Cdr1as-R | Mouse | Cdr1as | GGAAGATCACGATTGTCTGGAAG |
| m_cirTulp4-F | Mouse | circTulp4 | TCACTGTCGCAGAGATAGGAGT |
| m_cirTulp4-R | Mouse | circTulp4 | GGCACTTGATATGTTTGTTTTCC |
| m_18S_Taqman | Mouse | 18S rRNA | Mm04277571_s1 |
| h_miR-7-5p_Taqman | Human | miR-7-5p | 483061_mir |
| h_miR-30d-5p_Taqman | Human | miR-30d-5p | 478606_mir |

**Supplementary Table 2 – Basic Demographics for EMBARC and ANTARES cohorts.** Tables showing the Mean ±SD for both age and either HAMD-17 (for EMBARC) or MADRS (for ANTARES) scores. Numbers of male and female patients are also shown

| EMBARC (N = 91) | Mean | SD |
| --- | --- | --- |
| Age | 38.41 | 13.71 |
| HAMD-17 baseline | 18.41 | 4.43 |
|  |  |  |
| ANTARES (N = 35) | Mean | SD |
| Age | 38.43 | 13.75 |
| MADRS baseline | 31.00 | 7.09 |
|  |  |  |
|  | Females | Males |
| EMBARC | 65 | 26 |
| ANTARES | 16 | 19 |
